# Resilience of aerobic methanotrophs in soils; spotlight on the methane sink under agriculture

**DOI:** 10.1093/femsec/fiae008

**Published:** 2024-02-07

**Authors:** Jiyeon Lim, Helena Wehmeyer, Tanja Heffner, Meret Aeppli, Wenyu Gu, Pil Joo Kim, Marcus A Horn, Adrian Ho

**Affiliations:** Institute for Microbiology, Leibniz Universität Hannover, Herrenhäuser Str. 2, 30419 Hannover, Germany; Nestlè Research, Route du Jorat 57, CH 1000 Lausanne 26, Switzerland; Institute for Microbiology, Leibniz Universität Hannover, Herrenhäuser Str. 2, 30419 Hannover, Germany; Environmental Engineering Institute IIE-ENAC, Laboratory SOIL, Ecole Polytechnique Fédérale de Lausanne (EPFL), Valais Wallis, CH 1950 Sion, Switzerland; Environmental Engineering Institute IIE-ENAC, Laboratory MICROBE, Ecole Polytechnique Fédérale de Lausanne (EPFL), CH 1015 Lausanne, Switzerland; Division of Applied Life Science, Gyeongsang National University, Jinju 660-701, Republic of Korea; Institute for Microbiology, Leibniz Universität Hannover, Herrenhäuser Str. 2, 30419 Hannover, Germany; Nestlè Research, Route du Jorat 57, CH 1000 Lausanne 26, Switzerland

**Keywords:** cover cropping, disturbances, methane oxidation, methanotroph ecology, organic amendment, tillage

## Abstract

Aerobic methanotrophs are a specialized microbial group, catalyzing the oxidation of methane. Disturbance-induced loss of methanotroph diversity/abundance, thus results in the loss of this biological methane sink. Here, we synthesized and conceptualized the resilience of the methanotrophs to sporadic, recurring, and compounded disturbances in soils. The methanotrophs showed remarkable resilience to sporadic disturbances, recovering in activity and population size. However, activity was severely compromised when disturbance persisted or reoccurred at increasing frequency, and was significantly impaired following change in land use. Next, we consolidated the impact of agricultural practices after land conversion on the soil methane sink. The effects of key interventions (tillage, organic matter input, and cover cropping) where much knowledge has been gathered were considered. Pairwise comparisons of these interventions to nontreated agricultural soils indicate that the agriculture-induced impact on the methane sink depends on the cropping system, which can be associated to the physiology of the methanotrophs. The impact of agriculture is more evident in upland soils, where the methanotrophs play a more prominent role than the methanogens in modulating overall methane flux. Although resilient to sporadic disturbances, the methanotrophs are vulnerable to compounded disturbances induced by anthropogenic activities, significantly affecting the methane sink function.

## Teaser

Synthesis and conceptualization of the response of aerobic methanotrophs to sporadic (one-off), recurring, and compounded disturbances, and their role as a methane sink in soils under agriculture management.

## Introduction

Methane is a potent greenhouse gas (GHG), having a 34-fold higher heat retentive capacity in a 100-year time frame than carbon dioxide (IPCC 2019). Atmospheric methane has increased to ∼1857 ppm_v_ in 2018, a 2.6-fold hike since the preindustrial era (IPCC 2019, Saunois et al. 2020). The recent trend in methane growth is a cause for concern, exacerbating the impact of climate change (Etminan et al. 2016, Dean et al. 2018), and indicates the imbalance of methane sources and sinks whereby the rate of methane production is outpaced by consumption (Saunois et al. 2020). Indeed, the net methane flux is a balance of methane production and oxidation, catalyzed by the methanogenic archaea (anaerobic decomposition of organic matter) and methanotrophs, respectively (Conrad 2009, Kirschke et al. 2013, Guerrero-Cruz et al. 2021). Particularly in well-aerated soils (e.g. forest, upland agricultural soils, and pasture), the methane flux is governed more by the activity of the aerobic methanotrophs than the methanogens (Serrano-Silva et al. 2014, Meyer et al. 2017, Ho et al. 2019). Hence, disturbances, including agricultural practices, inflicted upon the methanotrophs will inevitably affect the methane sink function in these soils. Anthropogenic-associated methane emissions, also accounting for agriculture-derived methane, contributes up to 65% of the total methane emitted globally (Nazaries et al. 2013).

Nevertheless, some agricultural practices may have a comparably lower environmental footprint than others (Lehmann et al. 2020). To this end, regenerative agricultural practices, which approximate or imitate natural systems are thought to render beneficial effects to soils (see below discussion). While the impact of (regenerative) agricultural practices on nitrous oxide fluxes and the associated microorganisms, specifically in relation to different (bio-based or mineral) fertilization regimes have been relatively well-documented (Cayuela et al. 2014, Yoon et al. 2019, El-Hawwary et al. 2022), how methane and the aerobic methanotrophs are affected by these interventions remain fragmented. This may, in part, stem from the general assumption that agricultural soils become less important methane sinks after conversion from pristine environments (Le Mer and Roger 2001, Ho and Bodelier 2015, Tate 2015, Kaupper et al. 2020). Here, we aim to (i) conceptualize the resilience and response of the methanotrophs to sporadic (i.e. one-off disturbances, allowing recovery of activity/community composition), recurring, and compounded environmental/anthropogenic disturbances, and (ii) consolidate research findings on the impact of agriculture, with emphasis on regenerative practices, on the methane sink function *via* pairwise comparisons of agricultural soils with and without specific interventions (magnitude or % change of the capacity of the soil to consume methane is documented). Practice-based agricultural interventions and the outcomes of these interventions were documented in a literature survey. We compiled field management practices (namely, nontillage, nonchemical-based fertilization, and cover cropping; Table S1, Supporting Information) largely considered to be regenerative (Lehmann et al. 2020, Newton et al. 2020), and focused on the impact of these practices on the methane flux, and with respect to the methanotroph ecology, when available. This compilation is not intended to be exhaustive, but rather to capture the breadth of the results (adverse to stimulatory effects of the practices on soil methane sink), particularly under upland cropping system. Individual agricultural practices were considered given that we cannot unequivocally attribute the response of the methane flux to a specific agricultural practice where multiple approaches were simultaneously applied (i.e. synergistic effect, such as integrating livestock and crop farming; Newton et al. 2020).

## Key players of aerobic methane oxidation

Discoveries over the past two decades have broadened the known diversity of methanotrophs, particularly the anaerobic ones which were found able to couple anaerobic methane oxidation to a suite of electron acceptors, including iron, sulphate, nitrite, and manganese; the ecology, physiology, and potential applications of the anaerobic methanotrophs have recently been reviewed (In ’t Zandt et al. 2018, Guerrero-Cruz et al. 2021). On the other hand, the aerobic methanotrophs (henceforth, referred as methanotrophs) oxidize methane to methanol using oxygen as the primary electron acceptor with the enzyme methane monooxygenase (MMO), which can be present as a soluble (sMMO) or membrane-bound particulate (pMMO) form. While the vast majority of methanotrophs harbor the pMMO, the alphaproteobacterial methanotrophs *Methylocella* and *Methyloferula* possess only the sMMO (Theisen et al. 2005, Vorobev et al. 2011). In methanotrophs harboring both the pMMO and sMMO, copper regulates the relative expression of these enzymes, suppressing the sMMO, while stimulating the pMMO (Knapp et al. 2007, Trotsenko and Murrell 2008). The *pmoA* and *mmoX* gene, respectively encoding for a subunit of the pMMO and sMMO, are frequently targeted in culture-independent studies to characterize the methanotrophs in complex communities (e.g. Liebner and Svenning 2013, Cai et al. 2016, Wen et al. 2016, Karwautz et al. 2018).

Besides the canonical proteobacterial methanotrophs, acidophilic and thermophilic/thermotolerant methanotrophs belonging to Verrucomicrobia were discovered in geothermal springs, but have since been found to be widespread (Schmitz et al. 2021, Kaupper et al. 2021b, Hwangbo et al. 2023). Interestingly, a cave-dwelling putative methanotroph (*candidatus Mycobacterium methanotrophicum*) was recently discovered, belonging to Actinobacteria (van Spanning et al. 2022). The methanotrophs possess distinct carbon assimilation pathways and metabolic finesse (Trotsenko and Murrell 2008). While around 50%–60% of methane-derived carbon is assimilated into the cell (remaining methane is oxidized to carbon dioxide *via* dissimilatory methane oxidation) in most methanotrophs, some methanotrophs (e.g. alphaproteobacterial *Methylosinus*) derived a substantial amount of cell carbon (≥ 60%) from carbon dioxide (Yang et al. 2013, Dedysh and Knief 2018). Additionally, some methanotrophs (e.g. *Methylocella*, and specific *Methylocystis* species, but not all) are facultative, capable of growth on compounds containing carbon–carbon bonds (e.g. acetate, ethanol, and succinate), besides methane (Dedysh et al. 2005, Im et al. 2011, Dedysh and Knief 2018). Other characteristics which differentiate the methanotrophs include their distinct phospholipid fatty acid (PLFA) profiles (Ho et al. 2019). The metabolic flexibility of methanotrophs may reflect on their ecological traits, influencing their habitat preference (Ho et al. 2013a, Knief 2015, 2017).

In particular, the aerobic rather than the anaerobic methanotrophs were often documented to be the active and key methane-oxidizers in many methane-emitting terrestrial environments (Blazewicz et al. 2012, Ho et al. 2013a, Gao et al. 2022, Kaupper et al. 2022). Interestingly, these methanotrophs may also foster close interactions with photosynthetic organisms, widening their habitat range to micro-oxic or even anoxic environments (Raghoebarsing et al. 2005, Ho and Bodelier 2015, Milucka et al. 2015, Guerrero-Cruz et al. 2021). It follows that high methane-emitting environments (e.g. wastewater treatment systems, landfill cover, rice paddies, and peatlands) are hotspots for the methanotrophs. Noteworthy, methanotrophs possessing MMO with a low affinity to methane (i.e. high concentration of substrate is required to saturate the MMO) and hence, tend to thrive in methane hotspots, are typically referred to as “low-affinity” methanotrophs (e.g. Ho et al. 2013a). Conversely, methanotrophs oxidizing methane at (circum-) atmospheric methane levels are anticipated to possess the enzyme with a high affinity to methane (henceforth, referred as “high-affinity” methanotrophs; Knief and Dunfield 2005, Bissett et al. 2012). Although representing a relatively minor fraction of the total bacterial population being members of the rare biosphere (Bodelier et al. 2013), the “low-affinity” methanotrophs disproportionally contribute to the total soil carbon (i.e. methane-derived carbon 1%–2%; Sultana et al. 2022). While the majority of cultured methanotrophs are “low-affinity” methane-oxidizers, typically but not exclusively recovered from high methane-emitting environments, the “high-affinity” methanotrophs have, for a long time been identified based on their *pmoA* gene diversity and resisted isolation (Cai et al. 2016, Pratscher et al. 2018, Ho et al. 2019, Tveit et al. 2019). Traditionally, these “high-affinity” methanotrophs have been clustered in specific clades (e.g. upland soil clusters USC-α and USC-γ, respectively belonging to Alpha- and Gamma-proteobacteria, as well as Jasper Ridge clusters JR1, JR2, and JR3; Knief 2015). Recently, a novel methanotroph capable of high-affinity methane oxidation belonging to a genus thought to consist of “low-affinity” methanotrophs, *Methylocapsa gorgona* has been isolated in subarctic Norway (Tveit et al. 2019), blurring the distinction between “high-“ and “low-affinity” methanotrophs on the phylogenetic level. Along with this isolate, other members of the same genus, *Methylocapsa acidiphila* and *Methylocapsa aurea* have also been shown to grow on atmospheric methane (Tveit et al. 2019). Although *Methylotuvimicrobium buryatense* can oxidize methane at relatively low concentrations, these values are still above atmospheric levels (>200 ppm_v_ for *M. buryatense*), and *M. buryatense* did not exhibit growth below the threshold methane concentrations (He et al. 2023). Therefore, with the exception of *Methylocapsa* species (Tveit et al. 2019), the lack of traditional “high-affinity” methanotroph isolates (e.g. members of USC-α, USC-γ, and JR clusters) capable of oxidizing and grow on atmospheric methane makes interpretation of their physiological response to disturbances challenging. Much remains unknown of this elusive methanotroph group. Having different affinities to methane may influence methanotroph distribution in the environment, with the “low-affinity” methanotrophs being more prevalent in environments with a high methane availability (% range), typically acting as a methane biofilter at oxic–anoxic interfaces, while the “high-affinity” ones consume atmospheric methane in well-aerated upland soils (Singh et al. 2010). However, it should be noted that the distribution of the “low-affinity” and “high-affinity” methanotrophs is not mutually exclusive, and they may co-occur. For instance, “low-affinity” methanotrophs may become active following a rainfall event in well-aerated upland soils as methane exceeding atmospheric levels becomes available with increased anoxic niches resulting in stimulated methanogenesis (Shrestha et al. 2012, Ho et al. 2013b). The different affinities for methane may also determine the response and resilience of the methanotrophic groups to disturbances (see below discussion).

## Conceptualizing the resilience of the methanotrophic activity and aerobic methanotrophs to sporadic, recurring, and compounded disturbances

**Figure 1.**
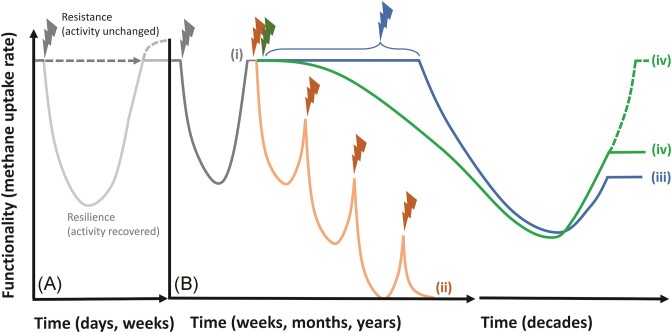
The effect of sporadic (A), recurring (B; i—grey line; ii—orange line), prolonged (B; iii—blue line), and compounded (B; iv—green line) disturbances on the methanotrophic activity (see Table S2, Supporting Information). In many instances, the recovery in methane uptake rates is not a reflection of the recovery in the methanotrophic community composition, indicating redundancy among the community members. Given sufficient recovery time under ample substrate (methane and oxygen) availability, methanotrophic activity typically recovers within days/weeks (light gray line; e.g. Pan et al. 2014, Kaupper et al. 2021a) or even over-compensate for initial activity loss (dashed light gray line; e.g. Ho and Frenzel 2012) likely attributable to higher nutrient and space availability (derived from disturbance-induced cell lysis and death) after sporadic disturbances (A). In (B), prior exposure to a disturbance may select for a seed bank community resistant to the disturbance for future contingencies. Hence, upon exposure to the same disturbance, activity will fully recover, and may even be less adversely affected (i—grey line; e.g. Krause et al. 2010, Baumann and Marschner 2013, van Kruistum et al. 2018). Without allowing a full recovery from prior disturbances, the methanotrophic activity eventually reached a “tipping point”, and thereafter, activity no longer recover with intensified recurring disturbance (ii—orange line; Ho et al. 2016, 2020). Following prolonged disturbances (iii—blue line), methanotrophic activity was profoundly altered, and did not recover to predisturbance levels (e.g. drought; Collet et al. 2015). Likewise, compounded disturbances (iv—green line) as expected under land-use change scenarios (i.e. peat mining, deforestation for agriculture; Tate 2015, Meyer et al. 2017, Reumer et al. 2018, Ho et al. 2022) significantly impaired the methanotrophic activity (particularly, “high-affinity” methane oxidation), but activity may return requiring extended recovery time spanning over decades (iv—dashed green line; e.g. Levine et al. 2011, McDaniel et al. 2019).

The “low-affinity” methanotrophs are remarkably resilient to sporadic or single disturbance events, having been shown to recover following a temperature and heat shock up to 45°C (Ho and Frenzel 2012), physical disruption to soil structure (sieving and grinding; Kumaresan et al. 2011), increasing salinity [soil salinity range 0.3–1.0 dS m^−1^, and up to saltwater salinity level (Bissett et al. 2012, Ho et al. 2018)], and disturbance-induced mortality [soil recolonization following disturbances (Ho et al. 2011, Pan et al. 2014, Kaupper et al. 2020)], among other anthropogenic-induced disturbances (e.g. contamination of heavy metals, and pollutants such as pharmaceuticals, pesticides, and chemical additives; see Table S2, Supporting Information; Semrau et al. 2010, Deng et al. 2011, Benner et al. 2015). Given sufficient recovery time (within days to weeks) and substrate (methane and oxygen) availability, the “low-affinity” methanotrophs even over-compensated for disturbance-induced activity and diversity loss (Fig. 1). Also, relevant factors restricting microbial growth (i.e. nutrients and space, as a result of disturbance-induced cell die-off) may become available following disturbances. Therefore, the modified edaphic properties may determine the success of the early colonizers, benefiting the fast-growing methanotrophs under these favorable conditions (Ho et al. 2017). A compositional shift is often detected after disturbance, suggesting the differential response of community members to the disturbance leading to an altered trajectory in community succession (Table S2, Supporting Information; Kumaresan et al. 2011, Andersen et al. 2013, Kaupper et al. 2021a). In particular, the alphaproteobacterial methanotrophs (*Methylosinus* and *Methylocystis*), which showed habitat preference for relatively oligotrophic environments (e.g. ombrotrophic peatlands and upland soils), appeared to be generally more resistant to disturbances (Dedysh 2011, Ho et al. 2013a, Knief 2015, 2017), while the fast-growing gammaproteobacterial methanotrophs (e.g. *Methylobacter, Methylosarcina*, and *Methylobacter*) are likely the rapid-responders and early colonizers (Ho et al. 2013a, Pan et al. 2014, Kaupper et al. 2020). This suggests advantageous ecological traits inherent to some methanotrophs, likely reflecting on their life strategies, which enabled their persistence and dominance during and after disturbances, respectively (see reviews Ho et al. 2013a, 2017, Krause et al. 2014).

The resilience of the “low-affinity” methanotrophs may be attributable to relatively high methane availability in their habitat, allowing rapid proliferation among the surviving community members after disturbances, in contrast to the “high-affinity” methanotrophs, which are restricted by substrate availability (atmospheric methane), limiting growth and the population size (Knief and Dunfield 2005, Kolb et al. 2005, Ho et al. 2019). Importantly, the resilience of the “low-affinity” methanotrophs can also be partly explained by previous exposure to the same disturbance or disturbances, which elicited a similar physiological response, prompting rapid recovery of a community which had survived the event (Krause et al. 2012, 2017, Baumann and Marschner 2013, van Kruistum et al. 2018). It stands to reason that a microbial community primed to a disturbance eliciting a specific physiological response will respond more rapidly should the event reoccur. Although activity recovery can be attributable to prior exposure to a disturbance, results indicate the marginal role of site history in conferring resilience to contemporary disturbances, particularly for the “low-affinity” methanotrophs. Regardless of the community composition, methanotrophs from deep lake sediments recovered just as rapidly as methanotrophs from a shallow lake and rice paddy soil from desiccation and heat stress, despite not having prior exposure to the disturbance nor harboring the same community members (Ho et al. 2016). Nevertheless, prior disturbances likely selected for a reservoir of (seed bank) community members that were resistant or were even favored by the disturbance (Krause et al. 2010, van Kruistum et al. 2018). This begs the question whether the resilience of the methanotrophs will be challenged in the face of (intensified) recurring, and compounded disturbances.

To this end, methane uptake rates were shown to recover after consecutive desiccation–rewetting cycles induced every 2 weeks, but activity was significantly impaired when desiccation–rewetting events intensified (shortened recovery time from 2 to 1 week; Ho et al. 2016) and the effect increased over stress cycles. This suggests that disturbances may exert a cumulative effect on the soil methane uptake over time, and that the resilience of the “low-affinity” methanotrophs may eventually reach a “tipping point” with recurring disturbances (e.g. increased frequency of desiccation–rewetting events; Table S2, Supporting Information), as demonstrated in other microbial systems (Veraart et al. 2012, König et al. 2018). Impaired methane uptake rates were accompanied by a compositional shift in the recovered methanotrophic community, favoring members of *Methylocystis* (Ho et al. 2016). Similarly, a step-wise increase in ammonium concentrations from 0.5 to 4.75 g l^−1^ (in 0.25–0.5 g l^−1^ increments) significantly impaired methanotrophic activity or lengthened the lag before the onset of activity, but methane uptake could still be detected at the highest application rate, indicating the emergence of an ammonium-tolerant methanotrophic community with continuous and gradual exposure to increasing ammonium levels (Qiu et al. 2008, López et al. 2019, Ho et al. 2020). Whereas an abrupt ammonium increase elicited a dose-dependent effect on the soil methane uptake, likely favoring the more ammonium-resistant methanotrophs (i.e. able to detoxify products of ammonium oxidation like hydroxylamine, nitrate, and nitrite) such as those belonging to gammaprobacteria (e.g. *Methylosarcina, Methylocaldum, Methylococcus*, and *Methyobacter* (Noll et al. 2008, Poret-Peterson et al. 2008, van Dijk et al. 2021). These studies demonstrate that intensified and recurring disturbances imposed a cumulative effect on the methanotrophic activity, and profoundly alter the community composition, with consequences for future disturbances.

As with recurring disturbances, methanotrophic activity is significantly affected by compounded disturbances (i.e. multiple stressors inflicted simultaneously), as would be anticipated during a natural disaster and under anthropogenic-related land-use change such as land conversion for agricultural purposes. Following a peatland forest fire, the potential to oxidize methane was significantly impaired, concomitant to significantly reduced methanotroph abundance even after 7 years postrecovery (Danilova et al. 2015). The conversion of pristine to arable lands exacerbates methane emissions (thereafter, see below for effects of specific agricultural practices on the methane sink function; see Table S1, Supporting Information). Particularly for well-aerated upland soils, heightened methane emission following land conversion can be attributable to the loss of the methane sink function (Tate 2015, Meyer et al. 2017, Kroeger et al. 2021, Obregon Alvarez et al. 2023), which is projected to take up to 80 years to recover after the abandonment of agriculture (Levine et al. 2011, McDaniel et al. 2019). Likewise, deforestation of tropical rainforests for palm oil production significantly lowered the capacity of the soil to oxidize methane, but activity gradually recovered over decades (> 30 years) under oil palm agriculture (Kaupper et al. 2020, Ho et al. 2022). Comparing the methane uptake rates in a pristine, actively mined, and abandoned peatlands under different restoration interventions, activity in the dammed peatland postexcavation recovered after > 15 years with the return of *Sphagnum*, but the community composition was significantly altered, and the network of interacting microorganisms became less complex and connected (Andersen et al. 2010, Putkinen et al. 2018, Reumer et al. 2018, Kaupper et al. 2021b). The recovery in activity after peat mining was, thus not reflected in the recovery of the microbial population, resulting in a shift in the trajectory of community succession over time. Nevertheless, community shifts postdisturbance in peatlands may not necessarily be unfavorable with regard to methane emissions, considering that the comparably poorly established methanogenic community may lower methane production after restoration (Juottonen et al. 2012). In contrast to sporadic disturbances, these examples highlight the vulnerability of the methanotrophs to compounded disturbances, significantly impairing methanotrophic activity, as well as inducing compositional changes to the community. A shift in the methanotrophic composition may alter the collective traits of the methane-oxidizing community, exerting an effect on community functioning (Ho et al. 2013a, Krause et al. 2014, Nijman et al. 2021), more pronounced under fluctuating environmental conditions.

## Anthropogenic activity affecting soil methane sinks; spotlight on agricultural practices

Agriculture expansion and intensification to meet the global food, feed, and biofuel demands pose a threat to soil processes worldwide, including methane consumption. Although land conversion to agriculture may adversely impact soil ecosystem function, specific agricultural management practices may leave a less severe imprint. To this end, regenerative farming has been perceived as agricultural management approaches, which have a relatively lower environmental impact on soil ecosystem functions than conventional agriculture, at times, even purported to reverse the impact of conventional agriculture (e.g. carbon stock accumulation). Considered “sustainable land management practices” by the Intergovernmental Panel on Climate Change (IPCC), regenerative agriculture has been heralded as an effective strategy for continuous sustainable crop production (IPCC 2019). Yet, the concept lacks a clear definition or has been defined differently by users, albeit the widespread usage of the term. Agricultural practices, which are frequently associated with regenerative farming include reducing/eliminating tillage, use of cover crops including green manure, and integrated farming (Table S1, Supporting Information; Newton et al. 2020). Other exclusionary measures include no or minimum synthetic fertilizer input or replacing these with bio-based or organic residues (Table S1, Supporting Information; Lehmann et al. 2020). The impact of these agricultural practices particularly on edaphic parameters, crop yield, as well as carbon dioxide and nitrous oxide emissions in relation to (in)organic fertilization have been relatively well-documented in recent work (see discussion below). Although methane turnover in wetland rice cultivation is well-studied (e.g. Krüger et al. 2001, Kimura et al. 2004, Shrestha et al. 2011, Lee et al. 2014, Li et al. 2021), the impact of agriculture on the methane sink and the associated methanotrophs in upland soils remain fragmented. In particular, the response of the methanotrophic community composition and abundances are pertinent to explain variation in the response of the methane sink to diverse agricultural practices (Shrestha et al. 2012, Judd et al. 2016).

## The impact of agricultural practices on the methane sink

Here, we elaborate on the effects of specific agricultural practices (i.e. nontillage, exclusion of chemical N fertilization or incorporation of bio-based residues, cover cropping) on the methane sink function, with emphasis on upland soils (Table S1, Supporting Information; Lehmann et al. 2020, Newton et al. 2020). Because of the wide range of organic or bio-based residues used in case studies relevant at the local- or regional-scale (e.g. oil palm kernel and husks, diverse aboveground crop residues; Kaniapan et al. 2021, Shinde et al. 2022), we focused on compost and biochar, which can be derived from various waste streams, as well as manure or digestate, a commonly applied bio-based fertilizer.

**Figure 2.**
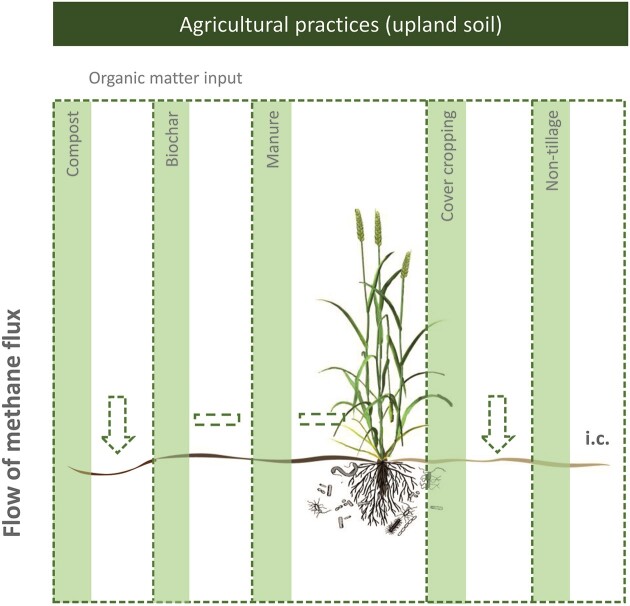
The impact of selected agricultural practices on methane emissions in well-aerated upland soils, comparing the effects of the treatments to agricultural soils without treatments (see Table S1, Supporting Information). The arrow indicates the direction of the change (increase or decrease); the magnitude of the change (%) is given in Table S1 (Supporting Information). Dashed outline indicates that the effect of an intervention has yet to be unambiguously resolved (e.g. potentially lower methane emissions following compost addition into upland agricultural soils). A dash indicates that the intervention imposed marginal or no change to methane emission. Abbreviations: i.c., inconclusive (insufficient studies to derive conclusion). Graphic of the crop is reproduced from Brenzinger et al. (2021).

The effects of tillage on soil methane emissions are contradictory, having been documented to significantly stimulate (e.g. Yeboah et al. 2016) or lower (e.g. Tian et al. 2013) methane uptake in agricultural soils (Fig. 2; Table S1, Supporting Information). This inconsistency may stem from the different types of cropping systems (wetland or well-aerated upland agriculture), exhibiting starkly different methane flux rates, in turn determining the magnitude and direction of fluxes (i.e. methane source or sink), and the response of the predominant indigenous methanotrophs (“low-affinity” or “high-affinity”) present. Similarly, the processes governing methane flux is different in the two cropping systems, with methanogenesis and anaerobic methane oxidation becoming important in the wetland soils. However, a general trend emerged when comparing the effects of nontillage and conventional tillage in wetland and upland agricultural soils independently, showing overall lower methane emission under nontillage in paddy fields (which may depend on the rice growing stage; Li et al. 2011), and having no apparent effects or lowered methane emission in upland agricultural soils (see review; Maucieri et al. 2021; Fig. 2; Table S1, Supporting Information). Comparatively lower methane emissions under nontillage in rice paddies are consistent with previous work (Huang et al. 2018). Rice paddies are commonly tilled between rice plants to remove weeds during the rice growing season. Tillage results in the aeration of soil and the oxidation of reduced electron acceptors, thereby providing thermodynamically favorable electron acceptors for microbial respiration and suppressing methanogenesis (Brune et al. 2000, Liesack et al. 2000). Moreover, tillage also disrupts the methane–oxygen counter gradient, which forms on the soil surface–overlaying floodwater interface (upper 1–3 mm, based on electrode measurements of substrate depth profiles), where the methanotrophs thrive. Here, the contribution of the methanotrophs to the net methane flux, typically determined using specific inhibitors, exhibited substantial methane consumption potentially up to 90% of total methane produced (Liesack et al. 2000, Kajan and Frenzel 2006, Reim et al. 2012, Prajapati and Jacinthe 2014). Hence, agricultural practices, which destroy this microhabitat will inevitably affect the role of the methanotrophs as a methane biofilter in rice paddies, requiring time (days to weeks; Ho et al. 2011) for the gradient and methanotroph population to re-establish. In contrast to wetland agriculture, tillage in well-aerated upland soils may act to relieve gas exchange limitation and promote methane uptake. When both nontilled and conventionally tilled upland agricultural soils act as methane sinks, atmospheric methane uptake can be lower in the nontilled than tilled site (Plaza-Bonilla et al. 2014), albeit the stimulatory effect of tillage could not be unambiguously confirmed in the presence of other confounding factors (Maucieri et al. 2021). Relevant local soil physico-chemical parameters, which may confound tillage-induced effects are moisture and temperature (Boeckx and Cleemput 1996, Hiltbrunner et al. 2012). Lower soil methane uptake in nontilled soils had been attributed to lower *in situ* temperature and high soil moisture in a field study, covering seasonal variation over a year (Tian et al. 2013), with lower temperature limiting biological activity including methane oxidation, whereas the high moisture content is thought to restrict gas (methane and oxygen) diffusion into the soil. While nontillage minimizes soil erosion and degradation, this intervention exerts different effects on soil methane emission, depending on the cropping system.

Another relevant agricultural practice that regenerates organic matter in soil is the exclusion and/or replacement of inorganic fertilizers with bio-based/organic residues (e.g. manure, as well as compost and biochar from diverse waste streams; Jenkinson 1991). However, the incorporation of bio-based organic residues, particularly manure, may still have undesirable side effects, including heightened methane emission *via* stimulation of the indigenous soil methanogens and/or the addition of residue-derived methanogens into the soil (Gattinger et al. 2007, Radl et al. 2007, Thangarajan et al. 2013,Ho et al. 2015). Manure-induced increase in methane emissions typically occur in rice paddies, while generally imposing little effect in upland agricultural soils (Fig. 2). Supplementing rice paddy soil with fresh manure promoted the soil-borne methanogens in flooded rice paddies, leading to significantly higher methane production (e.g. Kim et al. 2018), but can be remedied with the application of manure additives to the manure to suppress methane production, besides odor control (ammonia volatilization; Zhu 2000). Other bio-based residues show promising methane mitigation or crop growth-promoting capabilities; when locally sourced materials from diverse waste streams (e.g. agriculture, industry, and household) were applied to representative agricultural (sandy loam and clay) soils, some bio-based residues (e.g. nitrogen-rich sewage sludge and aquatic plant material) significantly increased crop (wheat) yield at the expense of having a higher global warming potential (GWP), mainly driven by nitrous oxide emissions (Ho et al. 2015, 2017). In the same study, the incorporation of compost in upland agricultural soils imposed comparably lower GWP than in the soils without any residue addition, and only marginally affected the soil bacterial community composition, including the methanotrophs, and fungal abundance (Ho et al. 2017, Brenzinger et al. 2018), in addition to promoting plant beneficial microbes (Bonanomi et al. 2018). Specific compost suppressed methane emission in well-aerated upland soils in the short-term (< 2 months) by significantly stimulating the apparent cell-specific methane uptake rates, offsetting up to 16% of the total carbon dioxide emitted (Ho et al. 2015, 2019, Brenzinger et al. 2018). Presumably, compost-derived rare earth metals (e.g. La and Ce) and other elements (e.g. copper and calcium) at minute concentrations (µg g soil^−1^ range; El-Ramady 2011) may have stimulated methanol dehydrogenase (catalyzes the conversion of methanol to formaldehyde) and/or the pMMO (in the case for copper) of some methanotrophs (Ho et al. 2013c, Zheng et al. 2018); Agegnehu et al. 2016, Vekeman et al. 2016, Krause et al. 2017). While methanotrophs may possess a copper sequestration mechanism by releasing methanobactin, a chalkphore with a high affinity for copper, and thus overcome copper limitation, a scavenging mechanism for the rare earth elements is as yet unknown in methanotrophs (Pol et al. 2014, DiSpirito et al. 2016). In contrast, compost induced significantly higher methane emission in wetland agricultural soils, considering high methane production under water-logged conditions. Despite having generally comparable physico-chemical properties (e.g. stable C fraction, or absence/minimal labile carbon), mature compost derived from different waste streams may differentially influence methane production and oxidation, affecting the overall flux (Brenzinger et al. 2018, van den Bergh et al. 2023). Hence, nuances in mature compost (e.g. presence of heavy metal contaminants or rare earth elements) may impose a strong effect on the soil methanotrophic community and activity. Although having no apparent effects on crop yield in these studies, compost amendment may thus reduce methane emissions and benefit other aspects of soil function (e.g. long-term carbon accumulation in soil; Ryals et al. 2015). Evidently, no improvement in crop yield was a trade-off for lower GWP, but the carbon dioxide offset by increased methane uptake suggests that crop productivity can be improved considering compost addition complemented with other N-rich soil additives (Brenzinger et al. 2021) at optimal combinations to minimize overall GHG emissions.

In addition to manure and compost, biochar application gained attention in the past decade, having been proposed as a carbon storage strategy in soils (Lehmann et al. 2006), and was projected to achieve carbon neutrality in agro-systems (rice, wheat, and corn production systems) when applied in combination with other climate-smart agricultural practices (intermittent drainage in rice production and reduced N-fertilization input; Xia et al. 2023). Although the effects of biochar amendments alongside conventional fertilizers on the edaphic properties have been well-documented (i.e. improved water and nutrient retention, cation exchange capacity, soil porosity, and aggregation leading to higher crop growth and yield; Liang et al. 2006, Mau and Utami 2014, Agegnehu et al. 2016, Bamminger et al. 2018, Rasa et al. 2018), the effects of biochar on GHG fluxes remain contentious. Biochar amendment can suppress or stimulate fertilizer-associated nitrous oxide emission (Yanai et al. 2007, Spokas et al. 2009, Cayuela et al. 2014, Harter et al. 2014, Shen et al. 2014, Agegnehu et al. 2016, Bamminger et al. 2018, Borchard et al. 2019). Similarly, what little is known on the effects of biochar on methane turnover is based on case studies, showing both a stimulation on methane production (e.g. Wang et al. 2012) and enhanced methane uptake (e.g. Karhu et al. 2011, Syed et al. 2016, Kubaczyński et al. 2022; Table S1, Supporting Information), as well as having no or marginal effects on methane emission (e.g. Bamminger et al. 2018). Like the effects of tillage, the apparent contrasting effects of biochar on the methane flux may stem from the cropping system, as well as the variable application rate in different studies (9–240 t ha^−1^; Spokas et al. 2009, Karhu et al. 2011, Bamminger et al. 2018, Zhao et al. 2021, Kubaczyński et al. 2022, Xia et al. 2023) and the delayed detectable effect over time (e.g. significant effects of biochar amendment detected only after 1 year; Major et al. 2010). Incorporation of biochar to wetland rice agricultural soils increased the methane sink strength or decreased the methane source when compared to amendments in upland agricultural soil, which showed marginal effects (Jeffery et al. 2016, Bamminger et al. 2018, Zhao et al. 2021). On the other hand, a recent study showed significant stimulation of methane uptake in upland agricultural soils concomitant to increased methanotroph abundance over at least 5 years after biochar addition (Kubaczyński et al. 2022). Moreover, biochar appeared to have a stabilizing effect, reducing the variability in methane fluxes (Karhu et al. 2011). Regardless of the feedstock (exception, biosolids) for biochar production, the pyrolysis temperature appears to be relevant in determining the effect of the final product on soil methane emission, with biochar undergone high pyrolysis temperature exceeding 600°C significantly increased the methane sink function after incorporation into soils (Jeffery et al. 2016). Biochar derived from high pyrolysis (> 600°C) contains less labile material (Bruun et al. 2011) and hence, less substrate availability for microorganisms (resistant to degradation), including the methanogens. Likewise, high porosity in biochar increases aeration, potentially suppressing methane production, or promotes methane oxidation (Karhu et al. 2011, Joseph et al. 2021). It thus appears that biochar modifies the edaphic properties, in turn, affecting microbially mediated soil processes; the direct effect of biochar, as well as other amendments, on methanotroph metabolism remains to be determined.

Besides no-tillage and incorporation of organic amendments into soils, regenerative farming includes cover cropping to minimize nitrogen loss *via* leaching and/or (de)nitrification in the presence of the main crops (intercropping) and during fallow after harvest (Pappa et al. 2011, Gabriel et al. 2012, Sanz-Cobena et al. 2014). Cover crops (e.g. legumes such as vetch and peas) may also be incorporated into the soil as green manure, thereby retaining accumulated N (i.e. having relatively slower mineralization rates; Baggs et al. 2000, Kim et al. 2012) in the field for the next cropping season. Also, depending on the selection of cover crops (mixtures or monocrop), substrate utilization profile assessed using a Biolog ECO plate analysis of soils amended with cover crop mixtures significantly increased, indicating a relatively higher microbial functional (metabolic) diversity when compared to soils that receive residues from monocrop (Drost et al. 2020). Species-specific effects of cover crops on carbon dioxide and nitrous oxide emissions have been documented, showing varied results (higher, lower, or comparable emission rates in fields without cover crops) for both intercropping and as green manure (Baggs et al. 2000, Pappa et al. 2011, Sanz-Cobena et al. 2014). However, the effects of cover cropping and green manure application on soil methane uptake are less known. Regardless of the choice of cover crops (barley, rape, and vetch), an upland agricultural soil planted to maize remained a methane sink, albeit having vetch as a cover crop turned the soil into a weak but not significant methane source during fallow (Sanz-Cobena et al. 2014). Like for carbon dioxide and nitrous oxide emissions (Sanz-Cobena et al. 2014, Drost et al. 2020), it appears that the C:N ratio of the cover crop is relevant when determining methane emissions. To this end, the choice of a cover crop as green manure in rice agriculture was shown to exert a strong effect on methane emission, with vetch possessing a lower C:N ratio resulting in significantly lower methane emission than rye (higher C:N), prompting the authors to suggest that the extraneous carbon (comparatively higher total C and labile C fractions) availability in rye upon incorporation into soil stimulated methanogenesis (Kim et al. 2012). Besides inducing a lower methane emission, vetch also significantly increased crop yield (total biomass and grain yield). Hence, a tailored selection of cover crops, also as green manure, for specific main crops and cropping systems are required to reduce methane emissions, while increasing yield. Evidently, future studies to explore the impact of cover cropping on methanotrophs are warranted.

## Conclusion and perspective

The methanotrophs are evidently affected by disturbances, but may still recover from sporadic events. Upon disturbance recurrence, however, methanotrophic activity was impaired, and required decades to recover following compounded disturbances associated to change in land use and natural disasters. Accumulating evidence indicates that the methane-oxidizing community is comprised of both methanotrophs and nonmethanotrophs, each play relevant roles, enabling and even exerting synergistic effects on community functioning (e.g. Stock et al. 2013, Ho et al. 2014, Benner et al. 2015, Veraart et al. 2018). Given the relevance of the nonmethanotrophs in modulating methanotrophic activity, future work could focus on interkingdom interaction in response to disturbances (incorporating soil micro- and macro-organisms e.g. viruses, protists, soil isopods; Murase and Frenzel 2008, Kuiper et al. 2013, Heffner et al. 2023a, b), and possibly, to establish early-warning indicators of a collapsing interaction network, leading to impaired community function. Moreover, interaction-induced release of (volatile) organic compounds can significantly influence the methanotrophs (Veraart et al. 2018), as well as the selection of beneficial microorganisms essential for crop protection (e.g. disease suppressive soils; Carrión et al. 2019, Weisskopf et al. 2021).

Although evidence suggests the transition to specific agricultural practices (e.g. nontillage, organic fertilization, and cover cropping) may favor or do not exert an adverse impact on the methanotrophs, applying such practices alone may not be sufficient to achieve food security for a growing human population. To this end, ecological intensification is generally thought to enhance soil ecosystem services by complementing and/or replacing conventional agricultural approaches to boost crop yields (Tittonell 2014, Kleijn et al. 2019, MacLaren et al. 2022). Central to ecological intensification is the enhancement of belowground (micro)organism interaction, which facilitates the usage of resources more efficiently. For instance, agricultural practices (e.g. low and sparse fertilization; Pandey et al. 2019) that favor dissimilatory nitrate reduction to ammonium over denitrification to retain N in soil (e.g. Putz et al. 2018, Yoon et al. 2019). Also, while the impact of specific agricultural practices on methane emissions and by extension, other parameters determining the multifunctionality of soils (e.g. physico-chemical characteristics, other GHG, microbial diversity) have been documented, the trade-off when applying multiple practices concurrently in conjunction with the individual practices, potentially yielding additive, synergistic, antagonistic, and/or net neural effects needs further probing (Lehmann et al. 2020, Xiao et al. 2021).

Emerging soil “modifiers,” such as nano- and microplastics are relatively persistent compounds, that not only alter soil characteristics, affecting gas diffusivity and the emissions/consumption of GHG, but also significantly affect the soil microbial (plastisphere; Rohrbach et al. 2022, Zhu et al. 2022) and invertebrate (e.g. earthworms and soil isopods; Lahive et al. 2022, Hink et al. 2023) communities. In addition, nanoplastics may accumulate in plants (Gong et al. 2021), and modify plant characteristics (e.g. change in root anatomy; Elena Pradas del Real et al. 2022), potentially affecting crop yield. Although the application of specific organic compounds such as biochar as soil additives has generally been well-received as a strategy to sequester carbon and immobilize heavy metals in soils (Gong et al. 2022), the environmental impact of long-term accumulation of the immobilized heavy metal remains unclear. The ambiguity of the long-term impact of these compounds (e.g. nanoplastics, microplastics, and biochar) in soils necessitates thorough environmental assessments. Summarized, regenerative agricultural practices can strengthen the methane sink and favor the methanotrophs, depending on the cropping system, but further work is needed to shed light on the mechanistic understanding of the outcomes of these agricultural practices.

## Supplementary Material

fiae008_Supplemental_File
