# Supplementary material for: Resilience of aerobic methanotrophs in soils; spotlight on the methane sink under agriculture
Source: FEMS Microbiol Ecol. 2024 Feb 7;100(3):fiae008. doi: 10.1093/femsec/fiae008 (PMC10872700; doi:10.1093/femsec/fiae008)
Supplement: fiae008_Supplemental_File [file fiae008_supplemental_file.docx]

Supplementary Materials

Resilience of aerobic methanotrophs in soils; spotlight on the methane sink under agriculture.

Jiyeon Lim^1^, Helena Wehmeyer^2^, Tanja Heffner^1^, Meret Aeppli^3^, Wenyu Gu^4^, Pil Joo Kim^5^, Marcus Horn^1^, Adrian Ho^2*^.

^1^Institute for Microbiology, Leibniz Universität Hannover, Herrenhäuser Str. 2, 30419 Hannover, Germany.

^2^Nestlè Research, Route du Jorat 57, CH 1000 Lausanne 26, Switzerland.

^3^Environmental Engineering Institute IIE-ENAC, Laboratory SOIL, Ecole Polytechnique Fédérale de Lausanne (EPFL), Valais Wallis, CH 1950 Sion, Switzerland.

^4^Environmental Engineering Institute IIE-ENAC, Laboratory MICROBE, Ecole Polytechnique Fédérale de Lausanne (EPFL), CH 1015 Lausanne, Switzerland.

^5^Division of Applied Life Science, Gyeongsang National University, Jinju 660-701, Republic of Korea.

*Corresponding author: Adrian Ho ([Adrian.HoKahWye@rd.nestle.com](mailto:Adrian.HoKahWye@rd.nestle.com)).

Included:

- Literature search criteria.
- Supplementary tables.

**Literature search criteria**

Supplementary Table S1 is based on a literature search in Google Scholar (<https://scholar.google.com>), according to the following criteria:

1. Research articles with empirical data were considered (e.g., modelling, life cycle assessments were excluded).
2. Pair-wise comparisons (with statistical analyses) of agricultural practices were considered. Research articles investigating the effects of multiple agricultural practices simultaneously (e.g., integrated farming systems) were excluded.
3. Research articles with study sites on croplands (i.e., wetland rice paddy and well-aerated upland agriculture) were considered. Other environments that are not typical of agricultural lands (e.g., saline rice paddies) were excluded.
4. Average values are given, comparing treatments to reference.
5. Keywords for the literature search were:

- Tillage/non-tillage: tillage, non-tillage, methane, methanogens, methanotrophs, methanogenic activity, and methane uptake.
- Organic matter input, manure: methane, organic matter, livestock manure, manure application, methanogens, methanotrophs, field study, arable soil.
- Organic matter input, compost: methane, organic matter, compost incorporation, methanogens, methanotrophs, field study, arable soil
- Organic matter input, digestate: methane, digestate, methanogens, methanotrophs, field study, arable soil.
- Organic matter input, biochar: methane, biochar, methanogens, methanotrophs, field study, arable soil.
- Cover cropping: methane, cover crop, green manure.

**Table S1**: Process-based agricultural practices that is regenerative, as outlined in Newton et al. (2020) and Lehmann et al. (2020). Outcome of agricultural practice with regard to methanotrophy (methane emission, methane oxidation potential, methanotroph community composition, if available) are listed. Only studies determining the effects of agricultural practices on net methane emissions and/or methane oxidation potential are listed.

| **Agricultural practice/management^a^** | **Geographical location** | **Wetland / non-wetland (well-aerated upland soils)**  **(crop)^b^** | **Application rate** | **Magnitude change CH_4_ flux (mean/range)** | **CH_4_ oxidation potential** | **Shift in microbial community abundances** | **Reference** |
| --- | --- | --- | --- | --- | --- | --- | --- |
| **Tillage / non-tillage** | Yujiang, China  (28°15’N, 116°55’E) | Wetland  (double-rice paddy) | n.a. | No significant difference in early rice season (tillage, 9.28 g m^-2^; non-tillage, 9.51 g m^-2^)  54 % lower CH_4_ emission in late rice season (tillage, 6.57 g m^-2^; non-tillage, 3.04 g m^-2^) | n.d. | n.d. | Li et al. (2011) |
|  | Yangzhou, China (32°350’N,  119°420’E) | Wetland  (rice-wheat rotation) | n.a. | No significant difference in wheat growing season (tillage, -0.82kg-C ha^-1^; non-tillage, -1.25 kg-C ha^-1^) | n.d. | n.d. | Yao et al. (2013) |
|  | Changshu, China  (31°330’N, 120°37’E) | Wetland  (rice-wheat rotation) | n.a. | 20 % higher CH_4_ emissions (averaged annual emissions in 3 years: tillage, 83.7 kg  ha^-1^; non-tillage, 100.1 kg ha^-1^) | n.d. | n.d. | Zhang et al. (2015) |
|  | Extremadura,  Spain  (39°06’N; 5°40’W) | Wetland  (rice paddy) | n.a. | 1^st^ year:  No significant difference (tillage, 322 kg ha^-1^; non-tillage, 206 kg ha^-1^)  2^nd^ year:  57 % lower CH_4_ emissions (tillage, 237 kg ha^-1^; non-tillage, 102 kg ha^-1^)  3^rd^ year:  87 % lower CH_4_ emissions (tillage, 500 kg ha^-1^; non-tillage, ~66 kg ha^-1^) | n.d. | n.d. | Fangueiro et al. (2017) |
|  | Wuxue,  China (29°55’N, 115°30’E) | Wetland  (rice paddy) | n.a. | 21 % lower CH_4_ emissions (tillage, ~66 g CH_4_ m^-2^; non-tillage, ~52 g CH_4_ m^-2^) | n.d. | n.d. | Ahmad et al. (2009) |
|  | Wuxue, China (29°510’N, 115°330’E) | Wetland  (rice paddy) | n.a. | 13 % lower CH_4_ emissions (tillage, ~292 kg ha^-1^; non-tillage, ~253 kg ha^-1^) | n.d. | n.d. | Zhang et al. (2016a) |
|  | Cachoeirinha, Brazil  (29.9°S,  51.1°W) | Wetland  (rice paddy) | n.a. | 21 % lower CH_4_ emissions (tillage, 517 kg CH_4_ ha^-1^; non-tillage, 408 kg CH_4_ ha^-1^) | n.d. | n.d. | Bayer et al. (2014) |
|  | Wuxue, China  (29°51’N, 115°33’E) | Wetland  (double-rice paddy) | n.a. | 53 % lower CH_4_ emissions in early season (tillage, ~64 g m^-2^; non-tillage, ~30 g m^-2^)  44 % lower CH_4_ emissions in late season (tillage, ~75 g m^-2^; non-tillage, ~42 g m^-2^) | n.d. | n.d. | Li et al. (2013) |
|  | Jinju,  South Korea  (35° 06’ 32.50” N, 128° 07’ 05.96” E) | Wetland  (rice paddy) | n.a. | 1^st^ year:  27 % lower CH_4_ emissions (tillage, 381 kg CH_4_ ha^-1^; non-tillage, 279 kg CH_4_ ha^-1^)  5^th^ year:  34 % higher CH_4_ emissions (tillage, 287 kg CH_4_ ha^-1^; non-tillage, 385 kg CH_4_ ha^-1^) | n.d. | 1^st^ year:  40 % lower  *mcrA* gene  copy numbers (tillage, 5.2x10^6^; non-tillage, 3.1x10^6^)  5^th^ year:  36 % higher  mcrA gene  copy numbers (tillage, 4.2x10^6^; non-tillage, 5.7x10^6^). | Kim et al. (2016) |
|  | Ningxiang, China  (112°18’E, 28°07’N) | Wetland  (double-rice paddy) | n.a. | Early season:  18 % lower CH_4_ emissions (tillage, ~228 kg CH_4_ ha^-1^; non-tillage, ~188 kg CH_4_ ha^-1^)  Late season:  4 % higher CH_4_ emissions (tillage, ~526 kg CH_4_ ha^-1^; non-tillage, ~506 kg CH_4_ ha^-1^) | n.d. | n.d. | Zhang et al. 2013) |
|  | Wuxue, China  (29°55’N, 115°30’E) | Wetland  (rice paddy) | n.a. | 1^st^ year:  49 % higher CH_4_ emissions (tillage, 6.76 g m^-2^; non-tillage, 4.54 g m^-2^)  2^nd^ year:  24 % higher CH_4_ emissions (tillage, 9.40 g m^-2^; non-tillage, 7.56 g m^-2^) | n.d. | n.d. | Cheng-Fang et al. (2012) |
|  | Beibei,  China  (30°26’N, 106°26’E) | Wetland  (rice paddy) | n.a. | No significant difference (tillage, 196 kg CH_4_ ha^-1^; non-tillage, 144 kg CH_4_ ha^-1^) | n.d. | n.d. | Hao et al. (2016) |
|  | Anding,  China  (35°28’N,  104°44’E) | Upland  (wheat-pea) | n.a. | No significant difference (average CH_4_ emissions: tillage, -0.098 mg m^-2^ h^-1^; non-tillage, -0.123 mg m^-2^ h^-1^) | n.d. | n.d. | Yeboah et al. (2016) |
|  | Agramunt,  Spain  (41°48’N, 1°07’E) | Upland  (maize) | n.a. | 18 % lower CH_4_ emissions (tillage, -0.55 kg CH_4_-C ha^-1^ yr^-1^; non-tillage, -0.65 kg CH_4_-C ha^-1^ yr^-1^) | n.d. | n.d. | Pareja-Sánchez et al. (2019) |
|  | Ohio,  USA  (41°0’37.8”N, 82°44’0.39”W) | Upland  (corn-wheat) | n.a. | No significant difference (tillage, 0.9 kg ha^-1^ y^-1^; non-tillage, 7.6 kg ha^-1^ y^-1^) | n.d. | n.d. | Elder & Lal (2008) |
|  | Agramunt,  Spain  (41°48’36”N, 1°07’06”E) | Upland  (barley) | n.a. | 57 % higher CH_4_ emissions in a short-term (tillage, -2.690 kg ha^-1^; non-tillage, -1.161 kg ha^-1^)  125 % lower CH_4_ emissions in a long-term (tillage, -1.065 kg ha^-1^; non-tillage, -2.396 kg ha^-1^) | n.d. | n.d. | Plaza-Bonilla et al. (2014) |
|  | Ohio,  USA  (39°45’N, 83°36’W) | Upland  (corn) | n.a. | 112-114 % lower CH_4_ emission  (tillage, 2.27-2.76 kg CH_4_-C ha^-1^ y^-1^;  non-tillage, -0.32 kg CH_4_-C ha^-1^ y^-1^) | n.d. | n.d. | Ussiri et al. (2009) |
|  | Madrid,  Spain  (40°32’N, 3°20’W) | Upland  (vetch & barley). | n.a. | No significant difference (tillage, ~-761 g CH_4_-C ha^-1^ y^-1^; minimum tillage, ~-891 g CH_4_-C ha^-1^ y^-1^; non-tillage, ~-581 g CH_4_-C ha^-1^ y^-1^) | n.d. | n.d. | Guardia et al. (2016) |
|  | Extremadura,  Spain  (39°19’06”N, 5°21’11”W) | Poorly drained upland soils  (triticale) | n.a. | 156 % lower CH_4_ emission (tillage, -2.73 mg CH_4_-C m^-2^; non-tillage, -6.98 mg CH_4_-C m^-2^) | n.d. | n.d. | García-Marco et al. (2016) |
|  | Ohio,  USA  (39°45’, 83°36’W),  (40°45’48”N, 81°54’20”W) | Upland  (corn) | n.a. | n.d. | 326 % higher CH_4_ oxidation (tillage, 0.061 µg CH_4_-C kg^-1^ h^-1^; non-tillage, 0.26 µg CH_4_-C kg^-1^ h^-1^) | n.d. | Prajapati & Jacinthe (2014) |
| **Organic matter input: manure** | Jinju,  South Korea  (35°06’N,  128°07’E) | Wetland  (rice paddy) | 5 dw t ha^-1^  (swine manure) | 180 % higher  CH_4_ emission  (+ manure,  ~26.7 t CO_2_-eq ha^-1^; - manure, ~9.5 t CO_2_-eq ha^-1^) | n.d. | n.d. | Jeong et al. (2018) |
|  | Beijing,  China | Wetland  (rice paddy) | ~1.1 t C ha^-1^  (pig manure)  ~1.1 t C ha^-1^  (cattle manure) | 3375 % higher mean CH_4_ emission  (+ manure,  139 mg m^-2^ d^-1^; - manure, 4 mg m^-2^ d^-1^)  675 % higher mean CH_4_ emission  (+ manure,  31 mg m^-2^ d^-1^; - manure, 4 mg m^-2^ d^-1^) | n.d. | n.d. | Wang et al. (2000) |
|  | Jinju,  South Korea | Wetland  (rice paddy) | 20 t ha^-1^  (cattle manure)  40 t ha^-1^  (cattle manure)  20 t ha^-1^  (swine manure)  40 t ha^-1^  (swine manure) | 94.5 % higher total CH_4_ emission  (+ manure,  ~10.7 mg m^-2^; - manure, ~5.5 mg m^-2^)  504 % higher total CH_4_ emission  (+ manure,  ~33.2 mg m^-2^; - manure, ~5.5 mg m^-2^)  45 % higher total CH_4_ emission  (+ manure,  ~8.0 mg m^-2^; - manure, ~5.5 mg m^-2^)  140 % higher total CH_4_ emission  (+ manure,  ~13.2 mg m^-2^; - manure, ~5.5 mg m^-2^) | n.d. | 615 % higher *mcrA* gene  copy numbers at panicle initiation stage (+ manure, ~186 x 10^6^ g dw soil^-1^; - manure, ~26 x 10^6^ g dw soil^-1^)  1335 % higher *mcrA* gene  copy numbers at panicle initiation stage (+ manure, ~373 x 10^6^ g dw soil^-1^; - manure, ~26 x 10^6^ g dw soil^-1^)  150 % higher *mcrA* gene copy numbers at panicle initiation stage (+ manure, ~65 x 10^6^ g dw soil^-1^; - manure, ~26 x 10^6^ g dw soil^-1^)  404 % higher *mcrA* gene  copy numbers at panicle initiation stage (+ manure, ~131 x 10^6^ g dw soil^-1^; - manure, ~26 x 10^6^ g dw soil^-1^) | Kim et al. 2014a) |
|  | Muñoz,  Philippines  (15°40’21”N, 120°53’26”E) | Wetland  (rice paddy) | 1.5 t ha^-1^  (chicken manure) | No significant difference (+ manure, 80 mg m^-2^ d^-1^; - manure, 79 mg m^-2^ d^-1^) | n.d. | n.d. | Corton et al. (2000) |
|  | Hiep Hoa,  Vietnam (21°20’N,  106°01E) | Wetland  (rice paddy) | 8 t ha^-1^  (pig manure) | 71 % higher CH_4_ emission (+ manure, 288 kg ha^-1^; - manure, 168 kg ha^-1^) | n.d. | n.d. | Vu et al. (2015) |
|  | Changshu,  China  (123°38’E, 31°33’N) | Wetland  (rice paddy) | 9 t ha^-1^  (pig manure compost) | 109 % higher CH_4_ emission (+ manure, ~65 g m^-2^; - manure, ~31 g m^-2^) | n.d. | n.d. | Chen et al. (2011) |
|  | New Delhi,  India  (28°40’N, 77°12’E) | Wetland  (rice paddy) | 30 kg N ha^-1^  (farmyard manure) | 39 % higher CH_4_ emission (+ manure, ~50 kg ha^-1^; - manure, ~36 kg ha^-1^) | n.d. | n.d. | Bhatia et al. (2005) |
|  | Nanjing,  China  (31°52’N,  118°50’E) | Wetland  (rice paddy) | 100 kg N ha^-1^  (farmyard manure) | 21 % higher CH_4_ emission (+ manure, ~127 kg ha^-1^; - manure, ~105 kg ha^-1^) | n.d. | n.d. | Qin et al. (2010) |
|  | New Delhi, India  (28°40’N, 77°12’E) | Wetland  (rice paddy) | 60 kg N ha^-1^  (farmyard manure) | 61 % higher CH_4_ emission (+ manure, ~45 kg ha^-1^; - manure, ~28 kg ha^-1^) | n.d. | n.d. | Pathak et al. (2003) |
|  | Cuttack,  India  (85°55’E, 20°25’N) | Wetland  (rice paddy) | 30 kg N ha^-1^  (poultry manure) | 23 % higher CH_4_ emission (+ manure, ~185 kg ha^-1^; - manure, ~150 kg ha^-1^) | n.d. | n.d. | Das & Adhya (2014) |
|  | Jinju,  South Korea  (35°06’N,  128°07’E) | Wetland  (rice paddy) | 5 dw t ha^-1^  (swine manure) | 181 % higher CH_4_ emission (+ manure, ~26.7 kg CO_2_-eq ha^-1^; - manure, ~9.5 kg CO_2_-eq ha^-1^) | n.d. | n.d. | Jeong et al. (2018) |
|  | Ludhiana,  India | Wetland  (rice paddy) | 20 t ha^-1^  (farmyard manure) | 419 % higher CH_4_ emission (+ manure, 111.6 kg ha^-1^; - manure, 21.5 kg ha^-1^) | n.d. | n.d. | Khosa et al. (2010) |
|  | Assam,  India  (26°41’N, 92°50’E) | Wetland  (rice paddy) | 5 t ha^-1^  (cow manure) | 45 % higher CH_4_ emission (+ manure, ~23 kg ha^-1^; - manure, 15.9 kg ha^-1^) | n.d. | n.d. | Bharali et al. (2018) |
|  | Wuhan,  China  (30°28’N, 114°25’E) | Wetland  (rice paddy) | 7 t ha^-1^  (pig manure)  11.6 t ha^-1^  (pig manure) | n.d. | n.d. | 857 % higher *mcrA* gene  copy numbers (+ manure, ~6.7 x 10^7^ g dw g-soil^-1^; - manure, ~0.7 x 10^7^ g dw g-soil^-1^)  No significant difference in *pmoA* gene  copy numbers (+ manure, ~1.3 x 10^7^ g dw g-soil^-1^; - manure, ~0.5 x 10^7^ g dw g-soil^-1^)  1471 % higher *mcrA* gene  copy numbers (+ manure, ~11 x 10^7^ g dw g-soil^-1^; - manure, ~0.7 x 10^7^ g dw g-soil^-1^)  No significant difference in *pmoA* gene  copy numbers (+ manure, ~1.1 x 10^7^ g dw g-soil^-1^; - manure, ~0.5 x 10^7^ g dw g-soil^-1^) | Zhang et al. (2018) |
|  | Harare, Zimbabwe (31°05’E,  18°11’S) | Upland  (rape) | 15 t ha^-1^  (cattle manure)  15 t ha^-1^  (cattle manure)  15 t ha^-1^  (cattle manure) | 1^st^ year:  No significant difference in average CH_4_ emission  (+ manure,  -0.2 g m^-2^ h^-1^; - manure, -0.1 mg m^-2^ h^-1^)  2^nd^ year:  No significant difference in average CH_4_ emission  (+ manure,  0.2 g m^-2^ h^-1^; - manure, 0.2 mg m^-2^ h^-1^)  3^rd^ year:  560 % higher average CH_4_ emission  (+ manure,  5.6 g m^-2^ h^-1^; - manure, 0.0 mg m^-2^ h^-1^) | n.d. | n.d. | Nyamadzawo et al. (2014) |
|  | Staffordshire, United Kingdom (52°47’43.9”N, 2°17’24.0”W) | Upland  (wheat-oat) | 40 t ha^-1^  (cattle manure) | No significant difference  (+ manure, ~7 mg CH_4_-C m^-2^;  - manure, ~-7 mg CH_4_-C m^-2^) | n.d. | n.d. | Rosace et al. (2020) |
|  | Jinxian,  China (28°37’N,  116°26’E) | Upland  (double-maize) | 1^st^ season in 2015:  15 t ha^-1^  (pig manure)  2^nd^ season in 2015:  15 t ha^-1^  (pig manure)  1^st^ season in 2016:  15 t ha^-1^  (pig manure)  2^nd^ season in 2016:  15 t ha^-1^  (pig manure) | 241 % higher CH_4_ emission  (+ manure, 1.88 kg ha^-1^;  - manure, -1.33 kg ha^-1^)  No significant difference  (+ manure, 0.06 kg ha^-1^;  - manure, -0.02 kg ha^-1^)  No significant difference  (+ manure, -0.93 kg ha^-1^;  - manure, -0.56 kg ha^-1^)  No significant difference  (+ manure, -0.65 kg ha^-1^;  - manure, -0.54 kg ha^-1^) | n.d. | n.d. | Afreh et al. (2018) |
|  | Wuwei,  China  (37°52’N, 102°50’ E) | Upland  (potato) | 1^st^ year:  200 kg N ha^-1^  (cattle manure)  2^nd^ year:  200 kg N ha^-1^  (cattle manure)  3^rd^ year:  220 kg N ha^-1^  (cattle manure) | No significant difference  (+ manure, -2.5 kg hm^-2^;  - manure, -1.2 kg hm^-2^)  48 % lower CH_4_ emission  (+ manure, -4.9 kg hm^-2^;  - manure, -3.3 kg hm^-2^)  No significant difference  (+ manure, -4.3 kg hm^-2^;  - manure, -3.4 kg hm^-2^) | n.d. | n.d. | Meng et al. (2020) |
|  | Yanting,  China  (31°16’N, 105°28’E) | Upland  (winter wheat)  Upland  (maize) | 52 kg N ha^-1^  (pig manure)  60 kg N ha^-1^  (pig manure) | No significant difference  (+ manure, -1.37 kg C ha^-1^;- manure, -1.43 kg C ha^-1^)  No significant difference  (+ manure, -0.62 kg C ha^-1^;  - manure, -0.7 kg C ha^-1^) | n.d. | n.d. | Zhou et al. (2014) |
|  | Madrid,  Spain  (40°32’N, 3°17’W) | Upland  (barley) | 125 kg N ha^-1^  (pig manure) | No significant difference  (+ manure, ~-229 mg C m^-2^;  - manure, -115 mg C m^-2^) | n.d. | n.d. | Meijide et al. (2010) |
|  | Lansing,  USA  (N 42.43,  W 84.28) | Upland  (corn-soybean) | 9.7 t ha^-1^  (cattle manure) | No significant difference (+ compost, 0.1 g CO_2_ m^-2^ y^-1^; - compost, -0.93 g CO_2_ m^-2^ y^-1^) | n.d. | n.d. | Thelen et al. (2010) |
| **Organic matter input: compost** | Jinju,  South Korea  (35°06’N,  128°07’E) | Wetland  (rice paddy) | 5 dw t ha^-1^  (composted swine manure) | 18 % higher  CH_4_ emission  (+ compost,  ~11.2 t CO_2_-eq ha^-1^; - compost, ~9.5 t CO_2_-eq ha^-1^) | n.d. | n.d. | Jeong et al. (2018) |
|  | Jinju,  South Korea | Wetland  (rice paddy) | 3 t ha^‑1^  (composted cattle manure) | 1^st^ year:  102 % higher  CH_4_ emissions (+ compost, ~545 kg ha^-1^; - compost, ~270 kg ha^-1^)  2^nd^ year:  No significant difference (+ compost, ~412 kg ha^-1^; - compost, ~375 kg ha^-1^) | n.d. | n.d. | Kim et al. (2014b) |
|  | Hiep Hoa,  Vietnam (21°20’N,  106°01E) | Wetland  (rice paddy) | 6.3 t ha^-1^  (Composted swine manure) | 29 % higher CH_4_ emission (+ compost, 217 kg ha^-1^; - compost, 168 kg ha^-1^) | n.d. | n.d. | Vu et al. (2015) |
|  | Changshu,  China  (123°38’E, 31°33’N) | Wetland  (rice paddy) | 9 dw t ha^-1^  (Composted swine manure) | No significant difference (+ compost, ~16 g m^-2^; - compost, ~31 g m^-2^) | n.d. | n.d. | Chen et al. (2011) |
|  | Changshu,  China  (31°33’N, 120°43’E) | Wetland  (rice paddy) | 180 kg N ha^-1^  (Composted swine manure) | 115 % higher CH_4_ emission (+ compost, ~131 kg C ha^-1^; - compost, ~61 kg C ha^-1^) | n.d. | n.d. | Wang et al. (2013) |
|  | Suzhou,  China  (31°32’N, 120°55’E) | Wetland  (summer rice)  Non-flooded (winter wheat) | 6 t ha^-1^  (Composted swine manure)  6 t ha^-1^  (composted swine manure) | No significant difference (+ compost, 98.8 kg C ha^-1^; - compost, 81.8 kg C ha^-1^)  65 % higher CH_4_ emission (+ compost, 18 kg C ha^-1^; - compost, 10.9 kg C ha^-1^) | n.d. | n.d. | Yang et al. (2015) |
|  | Jinju,  South Korea  (35°06’N,  128°07’E) | Wetland  (rice paddy) | 5 dw t ha^-1^  (composted swine manure) | No significant difference (+ compost, ~11.2 kg CO_2_-eq ha^-1^; - compost, ~9.5 kg CO_2_-eq ha^-1^) | n.d. | n.d. | Jeong et al. (2018) |
|  | Lelystad,  the Netherlands  (52°32’26.4”N, 05°33’34.7”E) | Upland  (wheat) | 20 t ha^-1^  (mature compost)  20 t ha^-1^  (wood shred-derived compost) | 19 % lower CH_4_ emission (+ compost, 70 µg kg soil^-1^; - compost, 83 µg kg soil^-1^)  6 % higher CH_4_ emission (+ compost, 88 µg kg soil^-1^; - compost, 83 µg kg soil^-1^) | n.d. | n.d. | Brenzinger et al. (2018) |
|  | The Netherlands, Vredepeel (51°32’32”N, 05°50’54”E) and  Lelystad (52°31’20”N, 05°34’57”E) | Upland  (wheat)  Upland  (wheat) | 20 t ha^-1^  (mature compost)  20 t ha^-1^  (mature compost) | 60 % lower CH_4_ emission  (+ compost, -5.36 mmol m^2^;  - compost, -2.12 mmol m^2^)  317 % lower CH_4_ emission (+ compost, 2.48 mmol m^2^; - compost, -5.38 mmol m^2^) | n.d. | n.d. | Ho et al. (2015) |
|  | Shin-Hidaka,  Japan  (42°260’N, 142°290’E) | Upland  (corn) | 1^st^ year:  40 fw t ha^-1^ (cattle manure compost)  2^nd^ year:  40 fw t ha^-1^ (cattle manure compost)  3^rd^ year:  40 fw t ha^-1^ (cattle manure compost) | No significant difference (+ compost, 0.5 kg ha^-1^; - compost, 5.1 kg ha^-1^)  No significant difference (+ compost, 0.4 kg ha^-1^; - compost, -0.2 kg ha^-1^)  No significant difference (+ compost, -0.7 kg ha^-1^; - compost, 1.9 kg ha^-1^) | n.d. | n.d. | Mukumbuta et al. (2017) |
|  | Lansing,  USA  (N 42.43,  W 84.28) | Upland  (corn-soybean) | 14.7 t ha^-1^  (dairy compost) | No significant difference (+ compost, -0.32 g CO_2_ m^-2^ y^-1^; - compost, -0.93 g CO_2_ m^-2^ y^-1^) | n.d. | n.d. | Thelen et al. (2010) |
| Organic matter input  : digestate (processed manure) | Hiep Hoa,  Vietnam (21°20’N,  106°01E) | Wetland  (rice paddy) | 65 kg N ha^-1^  (pig manure) | No significant difference (+ digestate, 162 kg ha^-1^; - digestate, 168 kg ha^-1^) | n.d. | n.d. | Vu et al. (2015) |
|  | Grugliasco,  Italy | Wetland  (rice paddy) | 800 kg C ha^-1^  (plant materials) | No significant difference (+ digestate, 493 kg ha^-1^; - digestate, 543 kg ha^-1^) | n.d. | n.d. | Bertora et al. (2020) |
|  | Yixing,  China  (31°26’N, 119°47’E) | Wetland  (rice paddy) | 1^st^ year:  120 kg N hm^-2^  (pig manure)  180 kg N hm^-2^  2^nd^ year:  120 kg N hm^-2^  (pig manure)  180 kg N hm^-2^ | No significant difference (+ digestate, ~20 g m^-2^; - digestate, ~18 g m^-2^)  No significant difference (+ digestate, ~21 g m^-2^; - digestate, ~18 g m^-2^)  No significant difference (+ digestate, ~44 g m^-2^; - digestate, ~42 g m^-2^)  19 % higher CH_4_ emission (+ digestate, ~50 g m^-2^; - digestate, ~42 g m^-2^) | n.d. | n.d. | Huang et al. (2014) |
|  | Lelystad,  the Netherlands  (52°32’26.4”N, 05°33’34.7”E) | Well-aerated upland soils | 20 t ha^-1^  (manure) | 7 % lower CH_4_ emission  (+ digestate, -77 µg kg soil^-1^;  - digestate, 83 µg kg soil^-1^) | n.d. | No significant difference in the copy number of bacterial, archaeal, and fungal 16S rRNA gene | Brenzinger et al. (2018) |
|  | Puebla, Mexico | Upland  (wheat) | 150 kg N ha^-1^  (pig manure) | 122 % higher CH_4_ emission  (+ digestate, 3.2 µg CO_2_ m^-2^;  - digestate, -14.5 µg CO_2_ m^-2^) | n.d. | n.d. | Pampillón-González et al. (2017) |
|  | Staffordshire, United Kingdom (52°47’43.9”N, 2°17’24.0”W) | Upland  (wheat-oat) | 250 kg N ha^-1^  (cattle slurry) | 2500 % higher CH_4_ emission  (+ digestate, ~168 mg CH_4_-C m^-2^;  - digestate, ~-7 mg CH_4_-C m^-2^) | n.d. | n.d. | Rosace et al. (2020) |
|  | Madrid,  Spain  (40°32’N, 3°17’W) | Upland  (barley) | 125 kg N ha^-1^  (pig slurry) | No significant difference  (+ digestate, -286 mg C m^-2^;  - digestate, -115 mg C m^-2^) | n.d. | n.d. | Meijide et al. (2010) |
| **Organic matter input: biochar** | Hwaseong, South Korea (37°13’22”N, 127°02’32”E) | Wetland  (rice paddy) | 2 t ha^‑1^  (rice chaff, at 600 ℃) | 34 % lower CH_4_ emission (cumulative CH_4_: + biochar, ~16 g CH_4_ m^-2^;  - biochar, ~11 g CH_4_ m^-2^) | n.d. | n.d. | Kim et al. (2017) |
|  | Changsha,  China  (113°19’52”E, 28°33’04”N) | Wetland  (double-rice paddy) | 7.5 t ha^-1^  (rice straw)  22.5 t ha^-1^ | No significant difference (+ biochar, 43 kg C ha^-1^; - biochar, 43 kg C ha^-1^)  No significant difference (+ biochar, 25 kg C ha^-1^; - biochar, 26 kg C ha^-1^) | n.d. | n.d. | Shen et al. (2014) |
|  | Changsha,  China  (113°19’52”E, 28°33’04”N) | Wetland  (double-rice paddy) | 24 t ha^‑1^  (wheat straw, at 500^o^C)  48 t ha^‑1^ | 43 % lower CH_4_ emission (+ biochar, 82 kg C ha^-1^; - biochar, 144 kg C ha^-1^)  33 % lower CH_4_ emission (+ biochar, 96 kg C ha^-1^; - biochar, 144 kg C ha^-1^) | n.d. | n.d. | Chen et al. (2018) |
|  | Mymensingh,  Bangladesh | Wetland  (rice paddy) | 1 t ha^-1^  (sugarcane bagasse, at 400-500 ^o^C) | No significant difference (+ biochar, 124 kg ha^-1^; - biochar, 130 kg ha^-1^) | n.d. | n.d. | Ali et al. (2013) |
|  | Changsha,  China  (113°19’52”E, 28°33’04”N) | Wetland  (double-rice paddy) | Early rice season:  24 t ha^-1^  (wheat straw, at 500℃)  48 t ha^-1^  late rice season:  24 t ha^-1^  48 t ha^-1^ | No significant difference (+ biochar, 24 kg C ha^-1^; - biochar, 26 kg C ha^-1^)  No significant difference (+ biochar, 14 kg C ha^-1^; - biochar, 26 kg C ha^-1^)  41 % lower CH_4_ emission (+ biochar, 42 kg C ha^-1^; - biochar, 71 kg C ha^-1^)  35 % lower CH_4_ emission (+ biochar, 46 kg C ha^-1^; - biochar, 71 kg C ha^-1^) | n.d. | n.d. | Liu et al. (2014) |
|  | Tsukuba, Japan  (36°12’N, 140°09’E) | Wetland  (rice paddy) | 10 t ha^‑1^  (rice husk, at 350-400℃)  20 t ha^‑1^  40 t ha^‑1^ | No significant difference (+ biochar, 45.8 g m^-2^; - biochar, 30.9 g m^-2^)  No significant difference (+ biochar, 43.2 g m^-2^; - biochar, 30.9 g m^-2^)  No significant difference (+ biochar, 46.1 g m^-2^; - biochar, 30.9 g m^-2^) | n.d. | n.d. | Koyama et al. (2015) |
|  | Hangzhou,  China  (30°22’N, 119°51’E) | Wetland  (rice paddy) | 2.8 t ha^-1^  (rice straw)  22.5 t ha^-1^ | 41 % lower CH_4_ emission (+ biochar, 16 g C m^-2^; - biochar, 27 g m^-2^)  52 % lower CH_4_ emission (+ biochar, 13 g C m^-2^; - biochar, 27 g m^-2^) | n.d. | Methanogen abundance decreased/no significant difference in methanotroph abundance.  Methanogen and methanotroph abundances decreased | Nan et al. (2020) |
|  | Hangzhou, China | Wetland  (rice paddy) | 45 t ha^-1^  (rice straw, at 500 ℃) | 97 % lower CH_4_ emission (+ biochar, ~5 mg kg^-1^; - biochar, ~171 mg kg^-1^) | n.d. | Tillering stage:  56 % higher methanogen abundance (+ biochar, ~6.3 x 10^7^ copies g^-1^; - biochar, ~4.0 x 10^7^ copies g^-1^)  200 % higher methanotrophs abundance (+ biochar, ~12 x 10^4^ copies g^-1^; - biochar, ~4.0 x 10^4^ copies g^-1^)  Heading stage:  No significant difference in methanogen abundance (+ biochar, ~14 x 10^7^ copies g^-1^; - biochar, ~7.9 x 10^7^ copies g^-1^)  532 % higher methanotrophs abundance (+ biochar, ~15.8 x 10^4^ copies g^-1^; - biochar, ~2.5 x 10^4^ copies g^-1^) | Han et al. (2016) |
|  | Hwaseong-si, South Korea (37°13’22”N, 127°02’32”E) | Wetland  (rice paddy) | 2 t ha^-1^  (rice chaff, at 600 ℃) | 34 % lower CH_4_ emission (+ biochar, ~10.5 g m^-2^; - biochar, ~15.9 g m^-2^) | n.d. | Mid-summer season:  1018 % higher methanogen abundance (+ biochar, ~5.1 x 10^8^ copies g^-1^; - biochar, ~4.6 x 10^7^ copies g^-1^)  172 % higher methanotrophs abundance (+ biochar, ~9.8x 10^8^ copies g^-1^; - biochar, ~3.6 x 10^8^ copies g^-1^) | Kim et al. (2017) |
|  | Nanjing,  China | Wetland  (rice paddy) | 25 t ha^-1^  (rice husks, at 450 ℃)  50 t ha^-1^ | No significant difference (+ biochar, 26.6 kg C ha^-1^; - biochar, 19.6 kg C ha^-1^)  46 % higher CH_4_ emission (+ biochar, 28.7 kg C ha^-1^; - biochar, 19.6 kg C ha^-1^) | n.d. | n.d. | Wang et al. (2012) |
|  | Nanjing,  China | Upland  (wheat) | 25 t ha^-1^  (rice husks, at 450 ℃)  50 t ha^-1^ | No significant difference (+ biochar, -0.22 kg C ha^-1^; - biochar, 0.34 kg C ha^-1^)  No significant difference (+ biochar, 0.78 kg C ha^-1^; - biochar, 0.34 kg C ha^-1^) | n.d. | n.d. | Wang et al. (2012) |
|  | Finland (67°08’N, 24°97’E) | Upland  (cereals) | 9 t ha^-1^  (birch, at 400^o^C) | 96 % higher CH_4_ uptake (cumulative CH_4_ uptake: + biochar, ~97 g CH_4_-C ha^-1^; - biochar, ~50 g CH_4_-C ha^-1^) | n.d. | n.d. | Karhu et al. (2011) |
|  | Kaiyang,  China  (27°00’N, 107°02’E) | Upland  (cabbage & carrot) | 26t ha^-1^  (rapeseed & maize straw, at 550 ℃)  64 t ha^-1^  128 t ha^-1^ | No significant difference (seasonal flux: + biochar, -82.9 mg m^-2^; - biochar, -91.2 kg ha^-1^)  33 % lower CH_4_ emission (+ biochar, -121.4 mg m^-2^; - biochar, -91.2 kg ha^-1^)  80 % lower CH_4_ emission (+ biochar, -164.2 mg m^-2^; - biochar, -91.2 kg ha^-1^) | n.d. | n.d. | Fang et al. (2016) |
|  | Nanjing,  China  (32°01’N, 118°52’E) | Upland  (vegetable) | 20 t ha^-1^  (wheat straw, at 400 ℃)  40 t ha^-1^ | No significant difference (+ biochar, ~23 kg C ha^-1^; - biochar, ~7 kg C ha^-1^)  No significant difference (+ biochar, ~34 kg C ha^-1^; - biochar, ~7 kg C ha^-1^) | n.d. | n.d. | Li et al. (2015) |
|  | Gwangyang,  South Korea | Upland  (Chinese cabbage) | 1 t ha^-1^  (barley straw, at 400 ℃)  3 t ha^-1^  5 t ha^-1^ | No significant difference (+ biochar, ~8 g m^-2^; - biochar, ~10 g m^-2^)  No significant difference (+ biochar, ~8 g m^-2^; - biochar, ~10 g m^-2^)  No significant difference (+ biochar, ~7 g m^-2^; - biochar, ~10 g m^-2^) | n.d. | n.d. | Kang et al. (2016) |
|  | Ohio,  USA (40°02’00”N, 83°02’30”W) | Upland  (soybean) | 7.5 t ha^-1^  (wood, at 650 ℃) | No significant difference (+ biochar, ~7 kg ha^-1^; - biochar, ~3 kg ha^-1^) | n.d. | n.d. | Mukherjee et al. (2014) |
|  | Shenyang,  China  (41°49’N, 123°33’E) | Upland  (maize) | 2.63 t ha^-1^  (maize stover, at 350-550 ℃) | No significant difference (+ biochar, -0.3 kg ha^-1^; - biochar, -0.19 kg ha^-1^) | n.d. | n.d. | Yang et al. (2017) |
|  | Shangqiu,  China  (34°32’N, 115°30’E) | Upland  (maize) | 20 t ha^-1^  (wheat straw, at 350-550 ℃)  40 t ha^-1^ | 67 % higher CH_4_ emission (+ biochar, -0.13 kg ha^-1^; - biochar, -0.39 kg ha^-1^)  72 % higher CH_4_ emission (+ biochar, -0.11 kg ha^-1^; - biochar, -0.39 kg ha^-1^) | n.d. | n.d. | Zhang et al. (2012) |
|  | Xinzhou,  China  (38°298’N,  112°72’E) | Upland  (maize) | 20 t ha^-1^  (wheat straw, at 450 ℃)  40 t ha^-1^ | No significant difference (+ biochar, -0.86 kg ha^-1^; - biochar, -0.63 kg ha^-1^)  No significant difference (+ biochar, -0.43 kg ha^-1^; - biochar, -0.63 kg ha^-1^) | n.d. | n.d. | Zhang et al. (2016b) |
|  | Stuttgart, Germany | Upland  (winter rapeseed / spring wheat) | 30 t ha^‑1^  (*Miscanthus* x *giganteus*, at 850℃) | 11 % higher CH_4_ uptake (cumulative CH_4_: + biochar, ~50 mg CH_4_-C m^-2^; - biochar, ~45 mg CH_4_-C m^-2^) | n.d. | n.d. | Bamminger et al. (2018) |
|  | Yangling,  China  (34°20’N, 108°24’E) | Upland  (wheat-maize) | 8 t ha^-1^  (wheat straw, at 350-550℃)  16 t ha^-1^ | No significant difference (+ biochar, -1.1 kg C ha^-1^; - biochar, -2.4 kg C ha^-1^)  No significant difference (+ biochar, -4.4 kg C ha^-1^; - biochar, -2.4 kg C ha^-1^) | n.d. | n.d. | Zhang et al. (2017) |
| **Cover cropping** | Jinju,  South Korea  (35°8’56.73”N, 128°5’46.27”E) | Wetland  (rice paddy) | 25 t ha^-1^  (milk vetch)  29 t ha^-1^  (rye) | 61 % higher CH_4_ emission  (NPK, 483 kg ha^-1^; + milk vetch, ~778 kg ha^-1^)  122 % higher  (NPK, 483 kg ha^-1^; + rye, ~1,072 kg ha^-1^) | n.d. | n.d. | Kim et al. (2013) |
|  | Milyang,  South Korea (36°36’N,  128°45’E) | Wetland  (rice paddy) | 10 fw t ha^-1^  (Chinese milk vetch)  20 fw t ha^-1^  (Chinese milk vetch)  40 fw t ha^-1^  (Chinese milk vetch) | 33 % higher CH_4_ emission (+ cover crop, ~519 kg ha^-1^; - cover crop, 390 kg ha^-1^)  96 % higher CH_4_ emission (+ cover crop, ~764 kg ha^-1^; - cover crop, 390 kg ha^-1^)  353 % higher CH_4_ emission (+ cover crop, ~1377 kg ha^-1^; - cover crop, 390 kg ha^-1^) | n.d. | n.d. | Lee et al. (2010) |
|  | Hunan,  China  (28°11’58”N, 113°04’47”E) | Wetland  (double rice cropping paddy) | 7.5 t ha^-1^  (rape) | 353 % higher CH_4_ emission (+ cover crop, 61.5 g m^-2^; - cover crop, 34.6 g m^-2^) | n.d. | n.d. | Tang et al. (2015) |
|  | Qiyang,  China  (26°45’N,  111°52’E) | Wetland  (double rice cropping paddy) | 2.1 dw t ha^-1^  (ryegrass)  1.3 dw t ha^-1^  (milkvetch)  1.2 dw t ha^-1^  (rapeseed) | 117 % higher CH_4_ emission (+ cover crop, ~2777 kg ha^-1^; - cover crop, ~1281 kg ha^-1^)  No significant difference (+ cover crop, ~1072 kg ha^-1^; - cover crop, ~1281 kg ha^-1^)  52 % higher CH_4_ emission (+ cover crop, ~1953 kg ha^-1^; - cover crop, ~1281 kg ha^-1^) | n.d. | n.d. | Raheem et al. (2019) |
|  | Cuttack,  India  (20°25’N, 85°55’E) | Wetland  (rice paddy) | 60 kg N ha^-1^  (Sesbania rostrata) | 57 % higher CH_4_ emission (+ cover crop, ~116 g m^-2^; - cover crop, ~74 g m^-2^) | n.d. | n.d. | Rath et al. (1999) |
|  | Ratchaburi,  Thailand | Wetland  (rice paddy) | 30 t ha^-1^  (Sesbania rostrata) | 57 % higher CH_4_ emission (+ cover crop, ~59 g C m^-2^; - cover crop, ~10 g C m^-2^) | n.d. | n.d. | Pimpan et al. (1994) |
|  | Jinju,  South Korea  (36°50’N,  128°26’E) | Wetland  (rice paddy) | 5.5 dw t ha^-1^  (barley)  4.3 dw t ha^-1^  (hairy vetch)  8.4 dw t ha^-1^  (barley:vetch=7.5:2.5) | 164 % higher CH_4_ emission (+ cover crop, 581 kg ha^-1^; - cover crop, 220 kg ha^-1^)  177 % higher CH_4_ emission (+ cover crop, 610 kg ha^-1^; - cover crop, 220 kg ha^-1^)  311 % higher CH_4_ emission (+ cover crop, 904 kg ha^-1^; - cover crop, 220 kg ha^-1^) | n.d. | n.d. | Hwang et al. (2017) |
|  | Jinju,  South Korea  (36°50’N,  128°26’E) | Wetland  (rice paddy) | 2.88 dw t ha^-1^  (barley:vetch=7.5:2.5)  5.76 dw t ha^-1^ (barley:vetch=7.5:2.5)  8.64 dw t ha^-1^ (barley:vetch=7.5:2.5)  11.5 dw t ha^-1^ (barley:vetch=7.5:2.5) | 79 % higher CH_4_ emission (+ cover crop, 367 kg ha^-1^; - cover crop, 205 kg ha^-1^)  369 % higher CH_4_ emission (+ cover crop, 961 kg ha^-1^; - cover crop, 205 kg ha^-1^)  519 % higher CH_4_ emission (+ cover crop, 1268 kg ha^-1^; - cover crop, 205 kg ha^-1^)  615 % higher CH_4_ emission (+ cover crop, 1466 kg ha^-1^; - cover crop, 205 kg ha^-1^) | n.d. | n.d. | Haque et al. (2013) |
|  | Jinju,  South Korea (35°8’56.5”N, 128°5’46.8”E) | Wetland  (rice paddy) | 1^st^ year:  5.1 fw t ha^-1^  (milk vetch)  5 fw t ha^-1^  (rye)  2^nd^ year:  25.8 fw t ha^-1^  (milk vetch)  29.4 fw t ha^-1^  (rye) | No significant difference (+ cover crop, 326 kg ha^-1^; - cover crop, 253 kg ha^-1^)  615 % higher CH_4_ emission (+ cover crop, 473 kg ha^-1^; - cover crop, 253 kg ha^-1^)  615 % higher CH_4_ emission (+ cover crop, 774 kg ha^-1^; - cover crop, 485 kg ha^-1^)  615 % higher CH_4_ emission (+ cover crop, 1069 kg ha^-1^; - cover crop, 485 kg ha^-1^) | n.d. | n.d. | Kim et al. (2012) |
|  | Madrid,  Spain  (40°03’N, 03°31’W) | Upland  (maize). | 180 kg ha^-1^  (barley)  150 kg ha^-1^  (vetch)  150 kg ha^-1^  (rape) | No significant difference (-0.01 - -0.03 kg CH_4_-C ha^-1^)  No significant difference (~-0.21 kg CH_4_-C ha^-1^)  No significant difference (-0.12 – 0.05 kg CH_4_-C ha^-1^) | n.d. | n.d. | Sanz-Cobena et al. (2014) |
|  | Staffordshire, United Kingdom (52°47’43.9”N, 2°17’24.0”W) | Upland  (oat) | n.d.  (fodder radish & vetch) | No significant difference  (+ cover crop, ~-2 mg CH_4_-C m^-2^;  - cover crop, ~-7 mg CH_4_-C m^-2^) | n.d. | n.d. | Rosace et al. (2020) |
|  | Madrid,  Spain  (40°03’N, 03°31’W) | Upland  (maize) | ~4.1 dw t ha^-1^  (barley)  ~2.1 dw t ha^-1^  (rape)  ~2 dw t ha^-1^  (vetch) | No significant difference (+ cover crop, -0.01 kg CO_2_-C ha^-1^; - cover crop, 0.05 kg CO_2_-C ha^-1^)  No significant difference (+ cover crop, -0.12 kg CO_2_-C ha^-1^; - cover crop, 0.05 kg CO_2_-C ha^-1^)  No significant difference (+ cover crop, -0.2 kg CO_2_-C ha^-1^; - cover crop, 0.05 kg CO_2_-C ha^-1^) | n.d. | n.d. | Sanz-Cobena et al. (2014) |
|  | Jinju,  South Korea  (35°146’N, 128°096’E) | Upland  (maize) | 1^st^ year:  10-11 fw t ha^-1^  (barley)  23-25 fw t ha^-1^  (hairy vetch)  2^nd^ year:  10-11 fw t ha^-1^  (barley)  23-25 fw t ha^-1^  (hairy vetch) | 677 % lower CH_4_ emission (+ cover crop, -1.73 kg ha^-1^; - cover crop, 0.3 kg ha^-1^)  No significant difference (+ cover crop, 0.77 kg ha^-1^; - cover crop, 0.3 kg ha^-1^)  1490 % lower CH_4_ emission (+ cover crop, -1.39 kg ha^-1^; - cover crop, 0.1 kg ha^-1^)  No significant difference (+ cover crop, 0.79 kg ha^-1^; - cover crop, 0.1 kg ha^-1^) | n.d. | n.d. | Cuello et al. (2015) |

Abbreviations: n.d., not determined; n.a., not applicable.

^a^Field management practices being promoted as, or are associated to regenerative agriculture.

^b^Cropping systems based on whether the soil is water saturated or inundated (i.e., wetland rice agriculture) and relatively well-aerated (i.e., upland agriculture).

**Table S2**: Response of the methanotrophic activity and community composition to sporadic, recurring, and compounded disturbances.

| **Disturbances**  **(environment)** | **Stressor mode** | **Response in methane uptake rate** | **Response in methanotrophic community composition**  **(methods to characterize methanotrophic composition)** | **References** |
| --- | --- | --- | --- | --- |
| **Sporadic disturbance (one-off stress events)** | | | | |
| Heat shock (rice paddy soil). | 37^o^C and 45^o^C for 42 h. | Full recovery after 6 days, relative to reference (incubations at 25^o^C). | Increased in alphaproteobacterial methanotrophs.  (qPCR and t-RFLP) | Ho and Frenzel (2012) |
| Drought – heat treatment (lake sediments and rice paddy soil). | Air-drying at 25^o^C (7 days) and 75^o^C (5 days), representing mild and severe stressor, respectively. | Full recovery after 26 days, relative to reference (fresh soil incubation). | Increased in alphaproteobacterial (*Methylocystis-/ Methylosinus*) and gammaproteobacterial (*Methylocaldum*- / Methylosarcina-related) methanotrophs.  (qPCR and diagnostic microarray) | Ho et al. (2016) |
| Drying-rewetting (rice paddy soil). | >95% gravimetric water loss by desiccation under the laminar flow cabinet at ~25^o^C for 15 hours. | Full recovery after 5 days, relative to reference (undisturbed soil). | Increased in alphaproteobacterial (*Methylocystis*).  (SIP coupled to Illumina MiSeq sequencing). | Kaupper et al. (2021a) |
| Soil physical disruption (landfill cover soil). | Sieving (2 mm mesh) and grinding (mortar and pestle). | Full recovery > 5 days to higher rates than in the un-disturbed soil. | Favored gammaproteobacterial (Methylobacter / Methylomonas / Methylomicrobium / Methylosarcina) methanotrophs.  (Diagnostic microarray). | Kumaresan et al. (2011) |
| Simulated disturbance-induced mortality (rice paddy soil). | Recolonization after disturbance (mixture of unsterilized soil in sterilized soil; soil sterilization by gamma-irradiation). | Full recovery > 4 days, relative to reference soil (unsterilized soil) | Increased in alphaproteobacterial methanotrophs (*Methylocystis / Methylosinus*).  (qPCR and diagnostic microarray). | Ho et al. (2011) |
| Simulated disturbance-induced mortality (river floodplain soil) | Recolonization after disturbance (mixture of unsterilized soil in sterilized soil; soil sterilization by gamma-irradiation and acetylene/kanamycin treatment). | Full recovery > 26 days, relative to reference soil (unsterilized soil). | Increased in alphaproteobacterial (*Methylocystis*) methanotrophs.  (qPCR and t-RFLP). | Pan et al. (2014) |
| NaCl-induced salt stress (rice paddy soil). | 0.005 – 0.6 M NaCl amendment (0.6 M NaCl, seawater salinity). | Resistant up to 0.3 M NaCl (methane uptake rate significantly lower > 0.3 M NaCl). | Favored gammaproteobacterial methanotroph (*Methylobacter*) up to 0.3 M NaCl.  (qPCR and Illumina MiSeq sequencing) | Ho et al. (2018) |
| Salinity gradient (woodlands). | 0.3 – 1.0 dS m-1 (low to high EC range). | N.d. | Increased relative abundance of alphaproteobacterial (*Methylocystis*) methanotrophs.  (Diagnostic microarray). | Bissett et al. (2012) |
| N loading (boreal wetlands) | NH_4_NO_3_ dose rate corresponding to 100 kg N ha^-1^. | No effect on methane uptake. | Favored gammaproteobacterial (*methylomonas / Methylobacter*), and decreased the relative abundances of alphaproteobacterial (*Methylocystis / Methylosinus*) methanotrophs.  (Diagnostic microarray). | Siljanen et al. (2012) |
| N loading (rice paddy soils). | Urea dose rates corresponding to 50-400 µg N g dw soil^-1^. | Resistant up to 200 µg N g dw soil^-1^ (methane uptake rate significantly lower > 200 µg N g dw soil^-1^). | Favored gammaproteobacterial (*Methylobacter / Methylomicrobium / Methylococcus*) methanotrophs.  (qPCR and DGGE). | Alam & Jia (2012) |
| N loading (landfill cover soil). | Dose rates corresponding to 0 (reference), 4, 20, 80 and 200 mM NH_4_. | Stimulation of activity, relative to reference. | Favored alphaproteobacterial (*Methylocystis* species) methanotrophs.  (Illumina MiSeq sequencing). | López et al. (2019) |
| N loading (rice paddy soil). | NH_4_Cl dose rates; 0.5 g L^-1^ to 4.75 g L^-1^. | Resistant up to 2.5 g NH_4_Cl L^-1^. | Favored gammaproteobacterial (*Methylosarcina*) methanotrophs.  (qPCR and Illumina MiSeq sequencing). | van Dijk et al. (2021) and references therein |
| Pollutant | Pyrene (polycyclic aromatic haydrocarbon, PAH) dose rate, 10-500 mg kg soil^-1^. | Resilient up to 22 mg pyrene kg soil^-1^. | Resistance of gammaproteobacterial (*Methylocaldum*) methanotroph at 500 mg pyrene kg soil^-1^.  (t-RFLP and cloning and sequencing). | Deng et al. (2011) |
| **Recurring disturbances** | | | | |
| Drying-rewetting cycles (rice paddy soil). | >94% gravimetric water loss by desiccation under the laminar flow cabinet at ~25^o^C for 16 hours, repeated weekly (severe stress) and fortnightly (moderate stress). | Fully recovered after moderate stress for 3 desiccation-rewetting cycles.  Not fully recovered after severe stress from the fifth desiccation-rewetting cycle onwards. | Predominance of type Ia (*Methylobacter*) methanotrophs during recovery.  Recovery of type II (*Methylocystis* / *Methylosinus*) methanotrophs over time.  (qPCR and t-RFLP). | Ho et al. (2016) |
| Drying-rewetting cycles, ammonium pulse cycles, and Drying-rewetting + ammonium pulse (riparian soil). | ~95% gravimetric water loss by desiccation under the laminar flow cabinet overnight, repeated weekly over 3 cycles.  NH_4_Cl_2_ dose rate corresponding to 50 mM soil porewater, repeated weekly over 3 cycles.  Drying-rewetting cycles, and introduction of an ammonium pulse at the final cycle. | Not fully recovered with each cycle of desiccation-rewetting.  Not fully recovered with each ammonium pulse.  Recovery (>7 days) with prior exposure to desiccation-rewetting cycles, following ammonium pulse. | Favored gammaproteobacterial (*Methylobacter*) methanotrophs.  Favored methylotrophs belonging to *Methylophilaceae*.  Favored gammaproteobacterial (*Methylobacter*) methanotrophs.  (qPCR and illumine MiSeq sequencing). | van Kruistum et al. (2018) |
| Intensified N loading (rice paddy soil). | Step-wise increase in NH_4_Cl concentrations (0.5 – 4.75 g L^-1^ at 0.25-0.5 g L^-1^ increments). | Significantly lowered methane uptake rate and increased lag before the onset of activity > 3.75 g NH_4_Cl L^-1^. | Favored alphaproteobacterial (*Methylocystis*) methanotrophs.  (qPCR and Illumina MiSeq sequencing). | Ho et al. (2020) |
| **Long-term/compounded disturbances** | | | | |
| Simulated long-term (1-18 years) drought (rice paddy soil). | Desiccated soils since 1 to 18 years. | Significantly lower methane uptake rates in soil experiencing 13-18 years, than in 1-5 years drought. | Recovery in type Ia (*Methylobacter*) methanotrophs.  (diagnostic microarray) | Collet et al. (2015) |
| Peat mining (peatlands). | >15 years post-excavation in restored peatlands. | Recovered methane uptake rate > 15 years (significantly lower methane uptake < 15 years after abandonment). | Predominance of alphaproteobacterial (*Methylocystis / Methylosinus*) methanotrophs.  (qPCR and t-RFLP). | Reumer et al. (2018);  Kaupper et al. (2021b) |
| Peat mining/extraction (peatlands). | 2, 17, and 63 years post-excavation in restored peatlands. | Recovered methane uptake rate > 17 years (lower methane uptake in the 2 and 17 year-old sites relative to pristine peatland). | Predominance of alphaproteobacterial (*Methylocystis*) methanotrophs. | Putkinen et al. (2018) |
| Wildfire (peatlands). | 7 years post-wildfire (burnt site). | Methane uptake rate did not recovered 7 years post-wildfire. | Significantly reduced methanotroph abundance.  Acidophilic type II methanotrophs replaced with less active type I methanotrophs in acidic bogs.  (cloning and sequencing). | Danilova et al. (2015) |
| Wildfire (peatlands). | 8-13 years post-wildfire (burnt site). | No significant change in the methane uptake rate compared to undisturbed peatland. | Predominance of *Methylomonas*-related methanotrophs.  (DGGE - cloning and sequencing). | Arai et al. (2014) |
| Deforestation (Rainforest soil) | Conversion of rainforest to agriculture (pasture). | N.d. | Decreased in alphaproteobacterial (*Methylocella / Methylosinus*) methanotrophs.  (Illumina HiSeq sequencing). | Meyer et al. (2017) |
| Land conversion (deciduous forest). | Conversion of forest to agriculture. | Significantly lower soil methane uptake, requiring ~80 years for full restoration (projected figure). | Predominance of putative “high-affinity” uncultured methanotrophs.  (PCR, cloning and sequencing). | Levine et al. (2011) |
| Land conversion (rainforest). | Conversion of forest to oil palm agriculture 7, 13, and 26 years post-conversion. | High-affinity methane oxidation did not recovered > 26 years post-conversion.  Recovery in low-affinity methane oxidation > 7 years post-conversion. | Predominance of puttive “high-affinity” uncultured methanotrophs.  (qPCR and Illumina MiSeq sequencing). | Ho et al. (2022) |

Abbreviations; EC, electrical conductivity; N.d., not determined; DGGE, denaturation gradient gel electrophoresis; qPCR, quantitative PCR; t-RFLP, terminal restriction fragment length polymorphism.

**References**

Afreh D, Zhang J, Guan D *et al.* Long-term fertilization on nitrogen use efficiency and greenhouse gas emissions in a double maize cropping system in subtropical China. *Soil Tillage Res* 2018;**180**:259–67.

Ahmad S, Li C, Dai G *et al.* Greenhouse gas emission from direct seeding paddy field under different rice tillage systems in central China. *Soil Tillage Res* 2009;**106**:54–61.

Alam MS, Jia Z. Inhibition of methane oxidation by nitrogenous fertilizers in a paddy soil. *Front Microbiol* 2012;**3**.

Ali MA, Hoque MA, Kim PJ. Mitigating global warming potentials of methane and nitrous oxide gases from rice paddies under different irrigation regimes. *Ambio* 2013;**42**:357–68.

Arai H, Hadi A, Darung U *et al.* A methanotrophic community in a tropical peatland is unaffected by drainage and forest fires in a tropical peat soil. *Soil Sci Plant Nutr* 2014;**60**:577–85.

Bamminger C, Poll C, Marhan S. Offsetting global warming-induced elevated greenhouse gas emissions from an arable soil by biochar application. *Glob Chang Biol* 2018;**24**:e318–34.

Bayer C, Costa F de S, Pedroso GM *et al.* Yield-scaled greenhouse gas emissions from flood irrigated rice under long-term conventional tillage and no-till systems in a Humid Subtropical climate. *Field Crops Res* 2014;**162**:60–9.

Bertora C, Moretti B, Peyron M *et al.* Carbon input management in temperate rice paddies: Implications for methane emissions and crop response. *Ital J Agron* 2020;**15**:144–55.

Bharali A, Baruah KK, Baruah SG *et al.* Impacts of integrated nutrient management on methane emission, global warming potential and carbon storage capacity in rice grown in a northeast India soil. *Environ Sci Pollut Res* 2018;**25**:5889–901.

Bhatia A, Pathak H, Jain N *et al.* Global warming potential of manure amended soils under rice-wheat system in the Indo-Gangetic plains. *Atmos Environ* 2005;**39**:6976–84.

Bissett A, Abell GCJ, Bodrossy L *et al.* Methanotrophic communities in Australian woodland soils of varying salinity. *FEMS Microbiol Ecol* 2012;**80**:685–95.

Brenzinger K, Drost SM, Korthals G *et al.* Organic residue amendments to modulate greenhouse gas emissions from agricultural soils. *Front Microbiol* 2018;**9**, DOI: 10.3389/fmicb.2018.03035.

Chen D, Wang C, Shen J *et al.* Response of CH4 emissions to straw and biochar applications in double-rice cropping systems: Insights from observations and modeling. *Environ Pollut* 2018;**235**:95–103.

Cheng-Fang L, Dan-Na Z, Zhi-Kui K *et al.* Effects of tillage and nitrogen fertilizers on CH_4_ and CO_2_ emissions and soil organic carbon in paddy fields of central China. *PLoS One* 2012;**7**.

Chen R, Lin X, Wang Y *et al.* Mitigating methane emissions from irrigated paddy fields by application of aerobically composted livestock manures in eastern China. *Soil Use Manag* 2011;**27**:103–9.

Collet S, Reim A, Ho A *et al.* Recovery of paddy soil methanotrophs from long term drought. *Soil Biol Biochem* 2015;**88**:69–72.

Corton TM, Bajita JB, Grospe FS *et al.* *Methane Emission from Irrigated and Intensively Managed Rice Fields in Central Luzon (Philippines)*. *Nutr. Cycl. Agroecosystems* 2000;**58**:37–53

Cuello JP, Hwang HY, Gutierrez J *et al.* Impact of plastic film mulching on increasing greenhouse gas emissions in temperate upland soil during maize cultivation. *Applied Soil Ecology* 2015;**91**:48–57.

Danilova O V., Belova SE, Kulichevskaya IS *et al.* Decline of activity and shifts in the methanotrophic community structure of an ombrotrophic peat bog after wildfire. *Microbiology (Russian Federation)* 2015;**84**:624–9.

Das S, Adhya TK. Effect of combine application of organic manure and inorganic fertilizer on methane and nitrous oxide emissions from a tropical flooded soil planted to rice. *Geoderma* 2014;**213**:185–92.

Deng H, Guo GX, Zhu YG. Pyrene effects on methanotroph community and methane oxidation rate, tested by dose-response experiment and resistance and resilience experiment. *J Soils Sediments* 2011;**11**:312–21.

Elder JW, Lal R. Tillage effects on gaseous emissions from an intensively farmed organic soil in North Central Ohio. *Soil Tillage Res* 2008;**98**:45–55.

Fang B, Lee X, Zhang J *et al.* Impacts of straw biochar additions on agricultural soil quality and greenhouse gas fluxes in karst area, Southwest China. *Soil Sci Plant Nutr* 2016;**62**:526–33.

Fangueiro D, Becerra D, Albarrán Á *et al.* Effect of tillage and water management on GHG emissions from Mediterranean rice growing ecosystems. *Atmos Environ* 2017;**150**:303–12.

García-Marco S, Abalos D, Espejo R *et al.* No tillage and liming reduce greenhouse gas emissions from poorly drained agricultural soils in Mediterranean regions. *Sci Total Environ* 2016;**566–567**:512–20.

Guardia G, Tellez-Rio A, García-Marco S *et al.* Effect of tillage and crop (cereal versus legume) on greenhouse gas emissions and Global Warming Potential in a non-irrigated Mediterranean field. *Agric Ecosyst Environ* 2016;**221**:187–97.

Han X, Sun X, Wang C *et al.* Mitigating methane emission from paddy soil with rice-straw biochar amendment under projected climate change. *Sci Rep* 2016;**6**.

Hao Q, Jiang C, Chai X *et al.* Drainage, no-tillage and crop rotation decreases annual cumulative emissions of methane and nitrous oxide from a rice field in Southwest China. *Agric Ecosyst Environ* 2016;**233**:270–81.

Haque MM, Kim SY, Pramanik P *et al.* Optimum application level of winter cover crop biomass as green manure under considering methane emission and rice productivity in paddy soil. *Biol Fertil Soils* 2013;**49**:487–93.

Ho A, Frenzel P. Heat stress and methane-oxidizing bacteria: Effects on activity and population dynamics. *Soil Biol Biochem* 2012;**50**:22–5.

Ho A, Lüke C, Frenzel P. Recovery of methanotrophs from disturbance: Population dynamics, evenness and functioning. *ISME Journal* 2011;**5**:750–8.

Ho A, Lüke C, Reim A *et al.* Resilience of (seed bank) aerobic methanotrophs and methanotrophic activity to desiccation and heat stress. *Soil Biol Biochem* 2016;**101**:130–8.

Ho A, Mendes LW, Lee HJ *et al.* Response of a methane-driven interaction network to stressor intensification. *FEMS Microbiol Ecol* 2020;**96**.

Ho A, Mo Y, Lee HJ *et al.* Effect of salt stress on aerobic methane oxidation and associated methanotrophs; a microcosm study of a natural community from a non-saline environment. *Soil Biol Biochem* 2018;**125**:210–4.

Ho A, Reim A, Kim SY *et al.* Unexpected stimulation of soil methane uptake as emergent property of agricultural soils following bio-based residue application. *Glob Chang Biol* 2015;**21**:3864–79.

Ho A, Zuan ATK, Mendes LW *et al.* Aerobic Methanotrophy and Co-occurrence Networks of a Tropical Rainforest and Oil Palm Plantations in Malaysia. *Microb Ecol* 2022;**84**:1154–65.

Huang HY, Cao JL, Wu HS *et al.* Elevated methane emissions from a paddy field in southeast China occur after applying anaerobic digestion slurry. *GCB Bioenergy* 2014;**6**:465–72.

Hwang HY, Kim GW, Kim SY *et al.* Effect of cover cropping on the net global warming potential of rice paddy soil. *Geoderma* 2017;**292**:49–58.

Jeong ST, Kim GW, Hwang HY *et al.* Beneficial effect of compost utilization on reducing greenhouse gas emissions in a rice cultivation system through the overall management chain. *Sci Total Environ* 2018;**613–614**:115–22.

Kang SW, Seo DC, Cheong YH., *et al*. Effect of barley straw biochar application on greenhouse gas emissions from upland soil for Chinese cabbage cultivation in short-term laboratory experiments. *J Mt Sci* 2016;**13**:693-702.

Karhu K, Mattila T, Bergström I *et al.* Biochar addition to agricultural soil increased CH­_4_ uptake and water holding capacity - Results from a short-term pilot field study. *Agric Ecosyst Environ* 2011;**140**:309–13.

Kaupper T, Mendes LW, Lee HJ. *et al*. When the going gets tough: emergence of a complex methane-driven interaction network during recovery from desiccation-rewetting. *Soil Biol Biochem* 2021a;**153**:108109.

Kaupper T, Mendes LW, Harnisz M *et al.* Recovery of Methanotrophic Activity Is Not Reflected in the Methane-Driven Interaction Network after Peat Mining. *Appl Environ Microbiol* 2021b;**87**:1–13.

Khosa MK, Sidhu BS, Benbi DK. *Effect of Organic Materials and Rice Cultivars on Methane Emission from Rice Field*. *J Environ Biol* 2010;**31**:281-285

Kim J, Yoo G, Kim D *et al.* Combined application of biochar and slow-release fertilizer reduces methane emission but enhances rice yield by different mechanisms. *Appl Soil Ecol* 2017;**117–118**:57–62.

Kim SY, Gutierrez J, Kim PJ. Considering winter cover crop selection as green manure to control methane emission during rice cultivation in paddy soil. *Agric Ecosyst Environ* 2012;**161**:130–6.

Kim SY, Gutierrez J, Kim PJ. Unexpected stimulation of CH_4_ emissions under continuous no-tillage system in mono-rice paddy soils during cultivation. *Geoderma* 2016;**267**:34–40.

Kim SY, Lee CH, Gutierrez J *et al.* Contribution of winter cover crop amendments on global warming potential in rice paddy soil during cultivation. *Plant Soil* 2013;**366**:273–86.

Kim SY, Pramanik P, Bodelier PLE *et al.* Cattle manure enhances methanogens diversity and methane emissions compared to swine manure under rice paddy. *PLoS One* 2014a;**9**.

Kim SY, Pramanik P, Gutierrez J *et al.* Comparison of methane emission characteristics in air-dried and composted cattle manure amended paddy soil during rice cultivation. *Agric Ecosyst Environ* 2014b;**197**:60–7.

Koyama S, Inazaki F, Minamikawa K *et al.* Increase in soil carbon sequestration using rice husk charcoal without stimulating CH4 and N2O emissions in an Andosol paddy field in Japan. *Soil Sci Plant Nutr* 2015;**61**:873–84.

Kumaresan D, Héry M, Bodrossy L *et al.* Earthworm activity in a simulated landfill cover soil shifts the community composition of active methanotrophs. *Res Microbiol* 2011;**162**:1027–32.

Lee CH, Park K Do, Jung KY *et al.* Effect of Chinese milk vetch (Astragalus sinicus L.) as a green manure on rice productivity and methane emission in paddy soil. *Agric Ecosyst Environ* 2010;**138**:343–7.

Levine UY, Teal TK, Robertson GP *et al.* Agriculture’s impact on microbial diversity and associated fluxes of carbon dioxide and methane. *ISME Journal* 2011;**5**:1683–91.

Li B, Fan CH, Zhang H *et al.* Combined effects of nitrogen fertilization and biochar on the net global warming potential, greenhouse gas intensity and net ecosystem economic budget in intensive vegetable agriculture in southeastern China. *Atmos Environ* 2015;**100**:10–9.

Li C, Zhang Z, Guo L *et al.* Emissions of CH_4_ and CO_2_ from double rice cropping systems under varying tillage and seeding methods. *Atmos Environ* 2013;**80**:438–44.

Li D, Liu M, Cheng Y *et al.* Methane emissions from double-rice cropping system under conventional and no tillage in southeast China. *Soil Tillage Res* 2011;**113**:77–81.

Liu J, Shen J, Li Y *et al.* Effects of biochar amendment on the net greenhouse gas emission and greenhouse gas intensity in a Chinese double rice cropping system. *Eur J Soil Biol* 2014;**65**:30–9.

López JC, Porca E, Collins G *et al.* Ammonium influences kinetics and structure of methanotrophic consortia. *Waste Manage* 2019;**89**:345–53.

Meijide A, Cárdenas LM, Sánchez-Martín L *et al.* Carbon dioxide and methane fluxes from a barley field amended with organic fertilizers under Mediterranean climatic conditions. *Plant Soil* 2010;**328**:353–67.

Meng C, Wang F, Yang K *et al.* Small wetted proportion of drip irrigation and non-mulched treatment with manure application enhanced methane uptake in upland field. *Agric For Meteorol* 2020;**281**.

Meyer KM, Klein AM, Rodrigues JLM *et al.* Conversion of Amazon rainforest to agriculture alters community traits of methane-cycling organisms. *Mol Ecol* 2017;**26**:1547–56.

Mukherjee A, Lal R, Zimmerman AR. Effects of biochar and other amendments on the physical properties and greenhouse gas emissions of an artificially degraded soil. *Sci Total Environ* 2014;**487**:26–36.

Mukumbuta I, Shimizu M, Hatano R. Mitigating global warming potential and greenhouse gas intensities by applying composted manure in cornfield: A 3-year field study in an Andosol soil. *Agriculture (Switzerland)* 2017;**7.**

Nan Q, wang C, Yi Q *et al.* Biochar amendment pyrolysed with rice straw increases rice production and mitigates methane emission over successive three years. *Waste Manage* 2020;**118**:1–8.

Nyamadzawo G, Wuta M, Nyamangara J *et al.* Nitrous oxide and methane emissions from cultivated seasonal wetland (dambo) soils with inorganic, organic and integrated nutrient management. *Nutr Cycl Agroecosyst* 2014;**100**:161–75.

PAMPILLÓN-GONZÁLEZ L, LUNA-GUIDO M, RUÍZ-VALDIVIEZO VM *et al.* Greenhouse Gas Emissions and Growth of Wheat Cultivated in Soil Amended with Digestate from Biogas Production. *Pedosphere* 2017;**27**:318–27.

Pan Y, Abell GCJ, Bodelier PLE *et al.* Remarkable Recovery and Colonization Behaviour of Methane Oxidizing Bacteria in Soil After Disturbance Is Controlled by Methane Source Only. *Microb Ecol* 2014;**68**:259–70.

Pareja-Sánchez E, Cantero-Martínez C, Álvaro-Fuentes J *et al.* Tillage and nitrogen fertilization in irrigated maize: key practices to reduce soil CO_2_ and CH_4_ emissions. *Soil Tillage Res* 2019;**191**:29–36.

Pathak H, Prasad S, Bhatia A *et al.* Methane emission from rice-wheat cropping system in the Indo-Gangetic plain in relation to irrigation, farmyard manure and dicyandiamide application. *Agric Ecosyst Environ* 2003;**97**:309–16.

Pimpan J, Jun M, Paiboon P *et al*. Methane emission from plots with differences in fertilizer application in Thai paddy fields. *Soil Sci Plant Nutr* 1994;**40**:63-71.

Plaza-Bonilla D, Cantero-Martínez C, Bareche J *et al.* Soil carbon dioxide and methane fluxes as affected by tillage and N fertilization in dryland conditions. *Plant Soil* 2014;**381**:111–30.

Prajapati P, Jacinthe PA. Methane oxidation kinetics and diffusivity in soils under conventional tillage and long-term no-till. *Geoderma* 2014;**230–231**:161–70.

Putkinen A, Tuittila ES, Siljanen HMP *et al.* Recovery of methane turnover and the associated microbial communities in restored cutover peatlands is strongly linked with increasing Sphagnum abundance. *Soil Biol Biochem* 2018;**116**:110–9.

Qin Y, Liu S, Guo Y *et al.* Methane and nitrous oxide emissions from organic and conventional rice cropping systems in Southeast China. *Biol Fertil Soils* 2010;**46**:825–34.

Raheem A, Zhang J, Huang J *et al.* Greenhouse gas emissions from a rice-rice-green manure cropping system in South China. *Geoderma* 2019;**353**:331–9.

Rath AK, Swain B, Ramakrishnan B *et al.* *Influence of Fertilizer Management and Water Regime on Methane Emission from Rice Fields*. *Agric Ecosyst Environ* 1999;**76**:99–107

Reumer M, Harnisz M, Lee HJ *et al.* Impact of peat mining and restoration on methane turnover potential and methane-cycling microorganisms in a northern bog. *Appl Environ Microbiol* 2018;**84.**

Rosace MC, Veronesi F, Briggs S *et al.* Legacy effects override soil properties for CO_2_ and N_2_O but not CH_4_ emissions following digestate application to soil. *GCB Bioenergy* 2020;**12**:445–57.

Sanz-Cobena A, García-Marco S, Quemada M *et al.* Do cover crops enhance N_2_O, CO_2_ or CH_4_ emissions from soil in Mediterranean arable systems? *Sci Total Environ* 2014;**466–467**:164–74.

Shen J, Tang H, Liu J *et al.* Contrasting effects of straw and straw-derived biochar amendments on greenhouse gas emissions within double rice cropping systems. *Agric Ecosyst Environ* 2014;**188**:264–74.

Siljanen HMP, Saari A, Bodrossy L *et al.* Effects of nitrogen load on the function and diversity of methanotrophs in the littoral wetland of a boreal lake. *Front Microbiol* 2012;**3**.

Tang H, Xiao X, Tang W *et al.* Effects of winter covering crop residue incorporation on CH_4_ and N_2_O emission from double-cropped paddy fields in southern China. *Environ Sci Pollut Res* 2015;**22**:12689–98.

Thelen KD, Fronning BE, Kravchenko A *et al.* Integrating livestock manure with a corn-soybean bioenergy cropping system improves short-term carbon sequestration rates and net global warming potential. *Biomass Bioenergy* 2010;**34**:960–6.

Ussiri DAN, Lal R, Jarecki MK. Nitrous oxide and methane emissions from long-term tillage under a continuous corn cropping system in Ohio. *Soil Tillage Res* 2009;**104**:247–55.

van Dijk H, Kaupper T, Bothe C *et al.* Discrepancy in exchangeable and soluble ammonium-induced effects on aerobic methane oxidation: a microcosm study of a paddy soil. *Biol Fertil Soils* 2021;**57**:873–80.

van Kruistum H, Bodelier PLE, Ho A *et al.* Resistance and Recovery of Methane-Oxidizing Communities Depends on Stress Regime and History; A Microcosm Study. *Front Microbiol* 2018;**9**.

Vu QD, de Neergaard A, Tran TD *et al.* Manure, biogas digestate and crop residue management affects methane gas emissions from rice paddy fields on Vietnamese smallholder livestock farms. *Nutr Cycl Agroecosyst* 2015;**103**:329–46.

Wang J, Chen Z, Ma Y *et al.* Methane and nitrous oxide emissions as affected by organic-inorganic mixed fertilizer from a rice paddy in southeast China. *J Soils Sediments* 2013;**13**:1408–17.

Wang J, Pan X, Liu Y *et al.* Effects of biochar amendment in two soils on greenhouse gas emissions and crop production. *Plant Soil* 2012;**360**:287–98.

Wang ZY, Xu YC, Li Z *et al.* *A Four-Year Record of Methane Emissions from Irrigated Rice Fields in the Beijing Region of China*. *Nutr Cycl Agroecosystems* 2000;**58**:55–63

Yang B, Xiong Z, Wang J *et al.* Mitigating net global warming potential and greenhouse gas intensities by substituting chemical nitrogen fertilizers with organic fertilization strategies in rice-wheat annual rotation systems in China: A 3-year field experiment. *Ecol Eng* 2015;**81**:289–97.

Yang X, Lan Y, Meng J *et al.* Effects of maize stover and its derived biochar on greenhouse gases emissions and C-budget of brown earth in Northeast China. *Environ Sci Pollut Res* 2017;**24**:8200–9.

Yao Z, Zheng X, Wang R *et al.* Nitrous oxide and methane fluxes from a rice-wheat crop rotation under wheat residue incorporation and no-tillage practices. *Atmos Environ* 2013;**79**:641–9.

Yeboah S, Zhang R, Cai L *et al.* Greenhouse gas emissions in a spring wheat–field pea sequence under different tillage practices in semi-arid Northwest China. *Nutr Cycl Agroecosyst* 2016;**106**:77–91.

Zhang A, Cheng G, Hussain Q *et al.* Contrasting effects of straw and straw–derived biochar application on net global warming potential in the Loess Plateau of China. *Field Crops Res* 2017;**205**:45–54.

Zhang A, Liu Y, Pan G *et al.* Effect of biochar amendment on maize yield and greenhouse gas emissions from a soil organic carbon poor calcareous loamy soil from Central China Plain. *Plant Soil* 2012;**351**:263–75.

Zhang D, Pan G, Wu G *et al.* Biochar helps enhance maize productivity and reduce greenhouse gas emissions under balanced fertilization in a rainfed low fertility inceptisol. *Chemosphere* 2016a;**142**:106–13.

Zhang HL, Bai XL, Xue JF *et al.* Emissions of CH_4_ and N_2_O under Different Tillage Systems from Double-Cropped Paddy Fields in Southern China. *PLoS One* 2013;**8**.

Zhang W, Sheng R, Zhang M *et al.* Effects of continuous manure application on methanogenic and methanotrophic communities and methane production potentials in rice paddy soil. *Agric Ecosyst Environ* 2018;**258**:121–8.

Zhang Y, Sheng J, Wang Z *et al.* Nitrous oxide and methane emissions from a chinese wheat-rice cropping system under different tillage practices during the wheat-growing season. *Soil Tillage Res* 2015;**146**:261–9.

Zhang ZS, Chen J, Liu TQ *et al.* Effects of nitrogen fertilizer sources and tillage practices on greenhouse gas emissions in paddy fields of central China. *Atmos Environ* 2016b;**144**:274–81.

Zhou M, Zhu B, Brüggemann N *et al.* N_2_O and CH_4_ emissions, and NO_3_^-^ leaching on a crop-yield basis from a subtropical rain-fed wheat-maize rotation in response to different types of nitrogen fertilizer. *Ecosystems* 2014;**17**:286–301.
